# Targeting intratumoral bacteria reverses MHC-I suppression and enhances nano-based chemoimmunotherapy in colorectal cancer

**DOI:** 10.1016/j.mmr.2026.100036

**Published:** 2026-05-07

**Authors:** Jing Feng, Yan-Hong Liu, Li-Ming Gong, Wen-Xuan Zhang, Chen-Fei Liu, Cong-Cong Xiao, Li-Qing Chen, Ming-Ji Jin, You-Yan Guan, Zhong-Gao Gao, Wei Huang

**Affiliations:** a State Key Laboratory of Bioactive Substance and Function of Natural Medicines, Department of Pharmaceutics, Institute of Materia Medica, Chinese Academy of Medical Sciences and Peking Union Medical College, Beijing 100050, China; b Beijing Key Laboratory of Key Technologies for Natural Drug Delivery and Novel Formulations, Department of Pharmaceutics, Institute of Materia Medica, Chinese Academy of Medical Sciences and Peking Union Medical College, Beijing 100050, China; c Department of Pharmacy, the Affiliated Suzhou Hospital of Nanjing Medical University, Suzhou Municipal Hospital, Gusu School of Nanjing Medical University, Suzhou 215000, Jiangsu, China; d Department of Urology, National Cancer Center, National Clinical Research Center for Cancer, Cancer Hospital, Chinese Academy of Medical Sciences and Peking Union Medical College, Beijing 100021, China

**Keywords:** Intratumoral bacteria, Clinical data reanalysis, Targeted nanotherapy, Colorectal cancer (CRC), Immune microenvironment remodeling, Autophagy, Apoptosis

## Abstract

**Background:**

Emerging evidence indicates that diverse bacteria colonize tumors and facilitate oncogenesis through multifaceted mechanisms, predominantly through immunosuppression of the tumor microenvironment (TME). *Fusobacterium nucleatum* (*F.n*) exemplifies this phenomenon in colorectal cancer (CRC), where it mediates immune evasion via tumor cell autophagy and upregulation of programmed death-ligand 1 (PD-L1). Effective strategies for combining precise elimination of intratumoral *F.n* with chemotherapy remain lacking.

**Methods:**

A targeted multimodal nano-based chemoimmunotherapy was engineered by co-loading hyaluronic acid (HA)-coated silver nanoparticles (Ag NPs) and paclitaxel (PTX) into tumor-targeting cationic liposomes (Cls). The combination was mechanistically evaluated for *F.n* eradication capacity, antitumor effects, and remodeling of the tumor immune microenvironment. Antitumor efficacy and safety were also evaluated *in vivo* using a tumor-bearing mouse model.

**Results:**

Intratumoral *F.n* subverted antitumor immunity through a dual mechanism, coupling major histocompatibility complex class I (MHC-I) degradation with PD-L1 upregulation. HA@Ag NPs/PTX Cls exerted synergistic antitumor effects through multimodal mechanisms. HA@Ag NPs/PTX Cls eliminated intratumoral bacteria, restoring microbial homeostasis, enhancing MHC-I antigen presentation, increasing production of tumor necrosis factor (TNF) and interferon (IFN), and downregulating PD-L1 to promote tumor immunorecognition. Combined with PTX-induced apoptosis, the nanotherapy inhibited both primary and metastatic tumors. Notably, bacterial clearance-triggered immune activation established long-term immunological memory, providing durable protection against tumor recurrence. These results highlighted the dual benefit of liposomes in addressing microbial dysbiosis and immune evasion in CRC.

**Conclusion:**

This innovative liposomal strategy disrupts the immunosuppressive TME and synergizes with a small-molecule chemotherapeutic agent to induce tumor cell apoptosis, representing a novel approach to potentiate cancer immunotherapy.

## 1 Background

The gut microbiome plays a pivotal role in human health and has been implicated in the pathogenesis of various diseases [1]. *Fusobacterium nucleatum* (*F.n*), a prevalent oncobiont, is thought to reach colorectal cancer (CRC) tumors via gut mucosal translocation and systemic dissemination [2]. Clinically, *F.n* promotes oncogenesis through several mechanisms, including virulence factors, chronic inflammation, bacterial dysbiosis, and immune evasion [3], [4]. Furthermore, a high abundance of *F.n* correlates with reduced abundance of beneficial bacteria, such as *Streptococcus thermophilus* and *Veillonella* spp., which counteract microbial dysbiosis in carcinogenesis [5]. By disrupting this beneficial bacteria, *F.n* not only alters the tumor microenvironment’s (TME) ecological composition but also amplifies immune suppression—both through depletion of beneficial bacteria and activation of its own immunosuppressive pathways [6].

Various therapeutic strategies to eliminate tumor-colonizing *F.n*, primarily antibiotics and bacteriophages, have been explored [7], [8], collectively establishing the balance of intratumoral bacteria as a critical determinant of tumor progression in CRC. However, antibiotic treatments are constrained by emerging resistance, while hypoxia and the acidity of the TME may affect bacteriophage activity. In contrast, silver nanoparticles (Ag NPs) can facilitate the effective elimination of bacteria through several physicochemical mechanisms, including membrane disruption, protein denaturation, and reactive oxygen species induction, reducing the development of resistance while maintaining favorable biocompatibility and physicochemical stability [9].

Based on the pivotal role of intratumoral bacteria in shaping the immunosuppressive tumor microenvironment, this study aims to develop a CD44-targeted cationic liposome (Cls) system that concurrently achieves bacterial clearance and chemotherapeutic synergy. By specifically eliminating tumor-colonizing *F.n* while restoring major histocompatibility complex class I (MHC-I) antigen presentation and blocking programmed death-ligand 1 (PD-L1)-mediated immunosuppression, this integrated nanotherapeutic strategy is designed to overcome the multiple immunosuppressive barriers in tumors in combination with paclitaxel (PTX)-induced tumor cell apoptosis.

## 2 Methods

### 2.1 Clinical information access

Clinical data obtained from the National Center for Biotechnology Information (NCBI) public repository (https://www.ncbi.nlm.nih.gov/; Accession: PRJNA811533) were regrouped and reanalyzed. All patients included in the analysis were diagnosed with CRC and were treatment naïve at the time of tumor surgical resection.

### 2.2 Animals and tumor modeling

Male BALB/c mice (aged 6–8 weeks, *n*=226) were obtained from Vital River Laboratory Animal Technology Co., Ltd. (Beijing, China). Animal experiments were performed under the guidelines of the Laboratory Animal Ethics Committee of the Institute of Materia Medica, Chinese Academy of Medical Sciences, and Peking Union Medical College (IMM-N-25-0338 and 00004435).

An orthotopic CRC model with *F.n*-infection was established by injecting mice with 50 μl of a suspension containing 1×10^7^ C26^luc^ cells (described below) between the intestinal epithelium and mucus layer (designated as day 0). From post-implantation days 2–5, mice received daily oral gavage of *F.n* suspension [0.5 ml, 1×10^8^ colony-forming units (CFU)/ml]. Treatment was initiated on day 7, with peritumoral administration of 3-methyladenine (3-MA; 30 mg/kg) once daily for 7 d and (PTX, 10 mg/kg) every three days for a total of three doses (days 7, 10, 13). All tumors were collected for analysis on day 16.

### 2.3 Preparation of HA@Ag NPs/PTX Cls

HA@Ag NPs/PTX Cls were prepared using the film dispersion method [10]. Briefly, 1.4 mg 2-dioleoyl-3-trimethylammonium propane (DOTAP), 8.7 mg 1,2-dioleoyl-SN-glycero-3-phosphoethanolamine (DOPE), 2.9 mg cholesterol, and 0.5 mg PTX were dissolved in 0.5 ml of an organic solvent mixture (trichloromethane:methanol=8:2 v/v) under sonication. The solvent was evaporated for 20 min in a 37 °C water bath to form a thin lipid film, which was hydrated with 2 ml of an aqueous Ag NPs solution (10 μg/ml) at 60 °C for 2 h under continuous agitation. The resulting suspension was sonicated using an ultrasonic cell crusher (100 W; 4 s on/4 s off; median sonication time: 15 min). HA (40 µl, 1 mg/ml) was then added dropwise to allow adsorption onto the Cls surface via electrostatic interaction, followed by vortexing for 30 s. Blank liposomes (liposomes without encapsulated Ag NPs and PTX) and other Cls were prepared similarly.

### 2.4 Cell lines

C26^luc^, CD44^−^ (Caco2), and CD44^+^ (C26) cells were obtained from Genomeditech Co., Ltd. (Shanghai, China). The C26^luc^ cell line is a derivative of C26 generated by stable transfection with a luciferase expression vector to enable constitutive bioluminescence. Both C26^luc^ and C26 cell lines were cultured in 1640 medium supplemented with 4.5 g/L D-glucose, 10% (v/v) fetal bovine serum, and 1% (v/v) antibiotic-antimycotic solution (Penicillin 10,000 U/ml; Streptomycin 10 mg/ml). For C26^luc^ cells, 0.5 μg/ml puromycin was included for selection. Cells were maintained at 37 °C in a humidified incubator with 5% CO₂ and 95% air. Caco2 cell line was cultured in Dulbecco’s modified Eagle medium (DMEM) medium supplemented with 4.5 g/L D-glucose, 10% (v/v) fetal bovine serum, and 1% (v/v) antibiotic-antimycotic solution (Penicillin 10,000 U/ml; Streptomycin 10 mg/ml). Routine mycoplasma testing was performed for all cell lines using Hoechst 33258 staining [11].

### 2.5 Preparation of a bacteria-infected tumor cell model *in vitro*

Aliquots of *F.n* cultures (logarithmic growth phase) were harvested from overnight anaerobic cultures by centrifugation (4000× *g*, 10 min), washed twice with phosphate-buffered saline (PBS), and resuspended in antibiotic-free 1640 culture medium. Bacterial suspensions were diluted to 1×10^8^ CFU/ml and added to C26 cells in culture plates for 4 h (37 °C, 5% CO₂) prior to subsequent assays [12].

### 2.6 *In vivo* antitumor efficiency

Tumor fluorescence was monitored beginning on day 5 post-tumor implantation. Mice were grouped based on initial tumor fluorescence intensity once signals reached 2×10^11^ photons/s. Liposomes were administered by oral gavage (0.4 ml/d) containing paclitaxel (PTX, 5 mg/kg) and silver nanoparticles (Ag NPs, 100 μg/kg). Fluorescence intensity and body weight were recorded every 4 d. On post-implantation day 16, mice were euthanized, and tumor size, tumor weight, and colorectal length were measured. Tumor tissues were sectioned for hematoxylin and eosin (H&E) and terminal deoxynucleotidyl transferase-mediated dUTP nick-end labeling (TUNEL) staining. Colorectal, liver, and lung tissue sections were also stained with H&E. Mesenteric lymph nodes and spleen were collected for immune cell profiling via flow cytometry (Control group: tumor-bearing *F.n*-infected mice; Blank group: tumor-bearing *F.n*-uninfected mice).

Tumor inhibition rate and volume were calculated as follows:

Inhibition rate (%)=(Weight_control_−Weight_treatment_)/(Weight_control_)×100%.

Tumor volume=0.5×L×W×W, where L and W represent the tumor length and width, respectively.

Tumor sections were also stained for bacteria using eubacteria probe 338 (EUB338) (excitation wavelength: 548 nm; emission wavelength: 562 nm).

### 2.7 *In vivo* rechallenge model

Rechallenge experiments were performed in two groups of mice: 1) survivors at > 40 d post modeling who had received HA@Ag NPs/PTX Cls therapy; and 2) PBS control-treated mice (vehicle control, *F.n*-infected tumor-bearing mice receiving initial tumor inoculation followed by PBS treatment). Briefly, 1×10^6^ C26 cells in 100 μl PBS were inoculated into the right inguinal lymph node. To evaluate immune responses, tumors and spleens were harvested at 48 h and 7 d post-inoculation, respectively. Single-cell suspensions were prepared and stained with fluorescently labeled antibodies for flow cytometry analysis. Cytokine levels were analyzed by immunohistochemical (IHC) staining: interleukin (IL)-2 in the spleen and tumor necrosis factor-α (TNF-α)/interferon-γ (IFN-γ) in tumor specimens. Tumors were collected, photographed, and measured with digital calipers. Tumor volume and growth inhibition rates were calculated relative to controls.

### 2.8 Sequencing analysis of intratumoral bacteria

Bacterial species identification and relative abundance quantification were performed by sequencing the 5 R 16S rDNA gene following multiplex polymerase chain reaction (PCR) amplification. Bacterial DNA was extracted from tumor tissues using the Qubit bacterial DNA extraction Kit. PCR amplification was performed on the 5 R 16S rDNA targeting five hypervariable regions (V2, V3, V5, V6, and V8). Amplicons were sequenced on Illumina MiSeq. Sequence analysis was performed using the Greengenes database as a reference.

### 2.9 Biosafety analysis

For *in vivo* safety analysis of HA@Ag NPs/PTX Cls, healthy mice were weight-matched and divided into two treatment groups: 5% dextrose solution (Blank group only treated with 5% dextrose solution via oral gavage) and HA@Ag NPs/PTX Cls. Following 15 d of daily oral gavage, mice were humanely euthanized, and major organs were collected for H&E staining. Blood samples were also obtained for serum biochemical analysis of aspartate aminotransferase (AST), alanine aminotransferase (ALT), blood urea nitrogen (BUN), and creatinine (CRE). Body weight was recorded throughout the study period. Long-term toxicity evaluation was conducted at 1×, 5×, and 10× therapeutic doses, with histopathological changes observed after 28 d of treatment.

Detailed descriptions of other standardized procedures and reagent information are provided in **Additional file 1: Methods**.

### 2.10 Statistical analysis

Statistical analyses were performed using Student’s *t*-test for two-group comparisons and one-way analysis of variance (ANOVA) followed by Tukey’s post-test for multiple-group comparisons. Data are presented as mean±standard deviation (SD). Statistical significance was defined as a *P*-value<0.05. The sample size calculation method is detailed in **Additional file 1: Methods**.

## 3 Results

### 3.1 *F.n* drives TME remodeling via autophagy upregulation in CRC

**Fig. 1:**
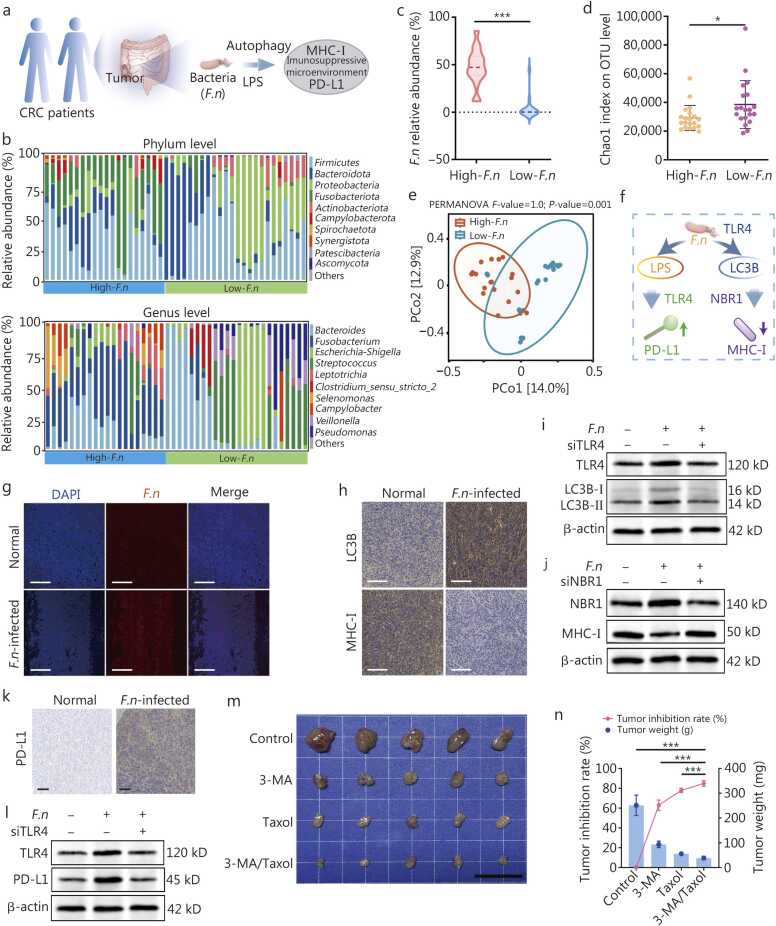
*F.n* drives tumor progression and remodels the tumor microenvironment via autophagy upregulation in CRC. **a** 16S rDNA gene data of intratumoral bacteria in clinical CRC patients downloaded from NCBI (PRJNA811533). **b** Stacked histograms indicating the relative abundance of bacterial communities at the phylum and genus levels in the clinical samples (high-*F.n* group, *n*=20; low-*F.n* group, *n*=24). **c** Violin plot showing *F.n* abundance (high-*F.n* group, *n*=20; low-*F.n* group, *n*=24). **d** Alpha diversity (Chao1 index) coefficient of intratumoral bacteria (high-*F.n* group, *n*=20; low-*F.n* group: *n*=24). **e** PCoA plot representing beta-diversity analysis of bacterial genera from each CRC tumor sample and PERMANOVA. **f** Schematic of *F.n*-driven immune microenvironment remodeling. **g** CLSM images of FISH, showing *F.n* infiltration in the *F.n*-infected model in mice. Scale bar=100 μm. **h** IHC staining of LC3B and MHC-I in tumor tissues. Scale bar=100 μm. **i** Western blotting analysis of LC3B expression in tumor cells co-cultured with *F.n*, with or without siRNA against TLR4. **j** Western blotting analysis of MHC-I expression in tumor cells co-cultured with *F.n*, with or without siRNA against NBR1. **k** IHC staining of PD-L1 in tumor tissues. Scale bar=100 μm. **l** Western blotting analysis of PD-L1 expression in tumor cells co-cultured with *F.n*, with or without TLR4 silencing. **m** Representative images of tumors treated with 3-MA and Taxol. **n** Tumor weight and tumor inhibition rate following 3-MA and Taxol treatment. Scale bar=2 cm (*n*=5). Data were presented as mean±SD, analyzed by Student’s *t*-test (**d, e**) and one-way analysis of variance followed by Tukey’s post-test (**o**). ^⁎^*P*<0.05, ^⁎⁎⁎^*P*<0.001. CRC. Colorectal cancer; MHC-I. Major histocompatibility complex class I; PD-L1. Programmed death-ligand 1; *F.n*. *Fusobacterium nucleatum*; DAPI. 4’,6-diamidino-2-phenylindole; TLR4. Toll-like receptor 4; LC3B. Microtubule-associated protein 1 A/1B-light chain 3B; LPS. Lipopolysaccharide; NBR1. Neighbor of the BRCA1 gene 1; PERMANOVA. Permutational MANOVA; 3-MA. 3-methyladenine; FISH. Fluorescence *in situ* hybridization; siRNA. Small interfering RNA; NCBI. National Center for Biotechnology Information; PCoA. Principal coordinates analysis; CLSM. Confocal laser scanning microscopy; IHC. Immunohistochemical.

Intratumoral pathogenic bacteria (especially *F.n*) suppress the tumor immune microenvironment and promote CRC progression via PD-L1/MHC-I-mediated immune suppression [6], [13] (Fig. 1**a**). Importantly, this pathogenic effect contrasts with the tumor-suppressive functions of certain probiotics, which exert antitumor activity through immune modulation [4]. Building upon the dual roles of intratumoral bacteria, we explored the role of intratumoral bacterial balance in tumor progression by reanalyzing 16S rDNA gene sequencing data from CRC patients in the clinical data obtained from the National Center for Biotechnology Information database. *F.n* was ubiquitous across all samples, with significant enrichment (≥45% relative abundance) observed in a subset of patients. Next, we stratified the samples into high- and low-*F.n* enrichment groups (Fig. 1**b, c**). Alpha diversity, reflecting species richness, was lower in the high-*F.n* group, whereas beta diversity, assessed via principal coordinates analysis (PCoA), revealed structural differences in the bacterial community between the groups. These results indicated that high-*F.n* abundance is associated with reduced bacterial diversity in tumors, thus affecting microbiome composition and balance (Fig. 1**d, e**). Multiple studies have reported that regions with *F.n* enrichment in colorectal tumors exhibit decreased CD4^+^ T cell infiltration alongside increased accumulation of immunosuppressive cell populations [6], [12]. Collectively, these findings demonstrated that pathogenic *F.n* bacteria promoted tumor growth by suppressing the immune microenvironment and the growth of beneficial microbes. Similarly, the growth of *Veillonella* spp.*,* which potentially promotes immune system maturation, was also suppressed in the high-*F.n* group (Fig. 1**b**).

Mechanistically, *F.n* appears to promote tumor immune evasion through downregulation of MHC-I and upregulation of PD-L1, collectively fostering an immunosuppressive TME (Fig. 1**f**). To validate this mechanism, we established an *F.n*-infected CRC mouse model. Immunofluorescence analysis confirmed successful bacterial colonization in tumors, validating model efficacy for subsequent studies (Fig. 1**g**).

Emerging evidence indicates that MHC-I molecules in tumor cells are selectively degraded by an autophagy-dependent mechanism, promoting immune evasion [14]. To assess whether intratumoral *F.n* modulates autophagy inhibition and MHC-I expression, IHC staining was performed for key autophagy pathway protein microtubule-associated protein 1 A/1B-light chain 3B (LC3B) and MHC-I in tumor tissues. *F.n* upregulated LC3B and downregulated MHC-I levels (Fig. 1**h; Additional file 1:** Fig. S1a), indicating activation of autophagy and reduced MHC-I-mediated antigen presentation by tumor cells. To investigate how *F.n* downregulates MHC-I expression, we explored the association between Toll-like receptor 4 (TLR4) TME signaling and autophagy in *F.n*-infected tumor cells. Western blotting analysis revealed that *TLR4* knockdown markedly reduced the *F.n-*induced expression of autophagy-related proteins, including LC3B, confirming TLR4 as a critical mediator of *F.n*-triggered autophagic activation (Fig. 1**i; Additional file 1:** Fig. S1b). Next, we investigated whether the autophagy receptor neighbor of *BRCA1* gene 1 (NBR1) mediates MHC-I degradation via autophagosomal targeting. *NBR1* knockdown with small interfering RNA (siRNA) upregulated MHC-I, indicating that NBR1-dependent autophagy constitutively suppresses MHC-I expression (Fig. 1**j; Additional file 1:** Fig. S1c**)**, consistent with previous reports [15], [16].

Bacterial surface lipopolysaccharide (LPS) reportedly upregulates PD-L1 expression on tumor cells [17], contributing to an immunosuppressive TME. To investigate this mechanism, we quantified PD-L1 expression on tumor tissues using IHC staining and enzyme-linked immunosorbent assay (ELISA). *F.n* infection significantly enhanced PD-L1 expression, promoting tumor immune escape via the PD-1/PD-L1 checkpoint axis. (Fig. 1**k; Additional file 1:** Fig. S1d). Next, we examined whether the TLR4-NF-кB axis mediates LPS-induced PD-L1 transcriptional activation in *F.n*-infected tumor cells. *TLR4* silencing using siRNA significantly attenuated *F.n*-induced PD-L1 upregulation, demonstrating that LPS enhances PD-L1 expression through a TLR4-NF-кB-dependent mechanism (Fig. 1**l; Additional file 1:** Fig. S1e).

Building on these findings, we inhibited *F.n*-induced autophagy with the inhibitor 3-MA and evaluated the antitumor efficacy of combining this treatment with Taxol, a first-line chemotherapeutic agent that stabilizes microtubules. Because apoptotic cells typically release insufficient immunostimulatory signals to mount an effective immune response [18], we hypothesized that combining autophagy inhibition with Taxol (the clinically approved formulation of PTX) could enhance antitumor effects. Indeed, the combination therapy significantly reduced tumor volume and tumor weight compared with either monotherapy (Fig. 1**m, n**), supporting a strategy that couples *F.n* elimination to alleviate autophagy with chemotherapy.

In summary, reanalysis of 16S rDNA sequencing data from CRC patient samples, combined with our mechanistic experiments, indicated that *F.n* can downregulate MHC-I via autophagy activation, while bacterial LPS enhances PD-L1 expression. This dual effect may impair tumor immune recognition, suggesting that immunotherapeutic strategies targeting *F.n* in combination with chemotherapy could improve antitumor efficacy in CRC.

### 3.2 Preparation and characterization of HA@Ag NPs/PTX Cls

Given the pivotal role of *F.n* in the immunosuppressive TME, we developed a targeted nano-based chemoimmunotherapeutic to remodel this microenvironment for CRC treatment. Cls-based nanocarriers are a current hotspot of biomaterials, combining good biocompatibility, simple structure, and scalable production, with a cationic surface charge that facilitates electrostatic-driven cellular uptake.

**Fig. 2:**
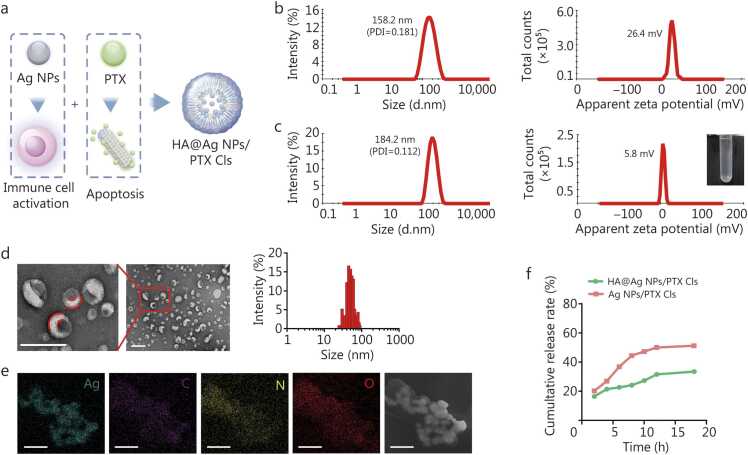
Characterization of HA@Ag NPs/PTX Cls. **a** Preparation methods and principles of HA@Ag NPs/PTX Cls. **b** Hydrodynamic particle size and zeta potential of Ag NPs/PTX Cls. **c** Hydrodynamic particle size and zeta potential of HA@Ag NPs/PTX Cls measured using DLS, with photographs of the HA@Ag NPs/PTX Cls suspension. **d** TEM images and histogram of HA@Ag NPs/PTX Cls. Scale bar=200 nm. **e** Elemental mapping of HA@Ag NPs/PTX Cls. Scale bar=500 nm. **f** Release profiles of HA@Ag NPs/PTX Cls and Ag NPs/PTX Cls in simulated gastric, intestinal, and colonic fluids *in vitro*. HA. Hyaluronic acid; Ag NPs. Silver nanoparticles; PTX. Paclitaxel; Cls. Cationic liposomes; PDI. Polydispersity index; DLS. Dynamic light scattering; TEM. Transmission electron microscopy.

A CD44-targeted drug delivery system was constructed by functionalizing Cls with HA to enable specific binding to CD44 receptors overexpressed on tumor cells, thereby enhancing cellular uptake [19]. Considering the synergistic interplay between immune microenvironment remodeling triggered by tumor-associated bacterial clearance and PTX-induced tumor cell apoptosis, Ag NPs and PTX were co-encapsulated within our HA-functionalized Cls to establish a precision co-delivery platform. The nanotherapy preparation process is shown in Fig. 2**a**. Water-soluble Ag NPs were encapsulated within the aqueous core, while hydrophobic PTX was loaded into the lipid bilayer. The prepared Cls were sonicated to ensure homogenous particle size and high encapsulation efficiency (EE) (Fig. 2**b; Additional file 1:** Tables S1, S2). Finally, anionic HA was electrostatically conjugated to the Cls surface through charge-mediated deposition.

Dynamic light scattering (DLS) analysis showed that Ag NPs/PTX Cls had a diameter of 152.8 nm [polydispersity index (PDI)=0.181] and surface zeta potential of 26.4 mV (Fig. 2**b**). Following HA coating, HA@Ag NPs/PTX Cls exhibited an increased particle size of 184.2 nm (PDI=0.112) and a reduced zeta potential of 5.8 mV (Fig. 2**c**). TEM imaging confirmed a core-shell nanostructure, comprising a hydrophilic core surrounded by a hydrophobic bilayer membrane (Fig. 2**d**). HA coating efficiency was quantified using a competitive binding assay with recombinant hyaluronan-binding protein [20], demonstrating complete encapsulating at 10,000.00 ng/ml HA (100% coating efficiency; **Additional file 1:** Table S3). Elemental mapping further confirmed Ag NPs incorporation within the liposomal matrix, as evidenced by the co-localization of Ag signals with characteristic liposomal elements (C, N, O) (Fig. 2**e**).

The EE% of Ag NPs and PTX in HA@Ag NPs/PTX Cls were quantified at 78.13% and 99.98%, respectively (**Additional file 1:** Tables S1, S2). To assess formulation stability, we conducted *in vitro* release studies under simulated gastrointestinal conditions. As shown in Fig. 2**f**, cumulative release of HA@Ag NPs/PTX Cls and Ag NPs/PTX Cls reached 16.45% vs*.* 20.23% in gastric fluid (2 h), 20.84% vs*.* 36.74% in intestinal fluid (2–6 h), and 33.47% vs*.* 51.26% in colonic fluid (6–18 h), respectively. These results demonstrated that HA surface modification enhanced NPs stability throughout simulated gastrointestinal transit.

### 3.3 HA-modified Cls exhibit superior tumor uptake *in vitro* and *in vivo*

**Fig. 3:**
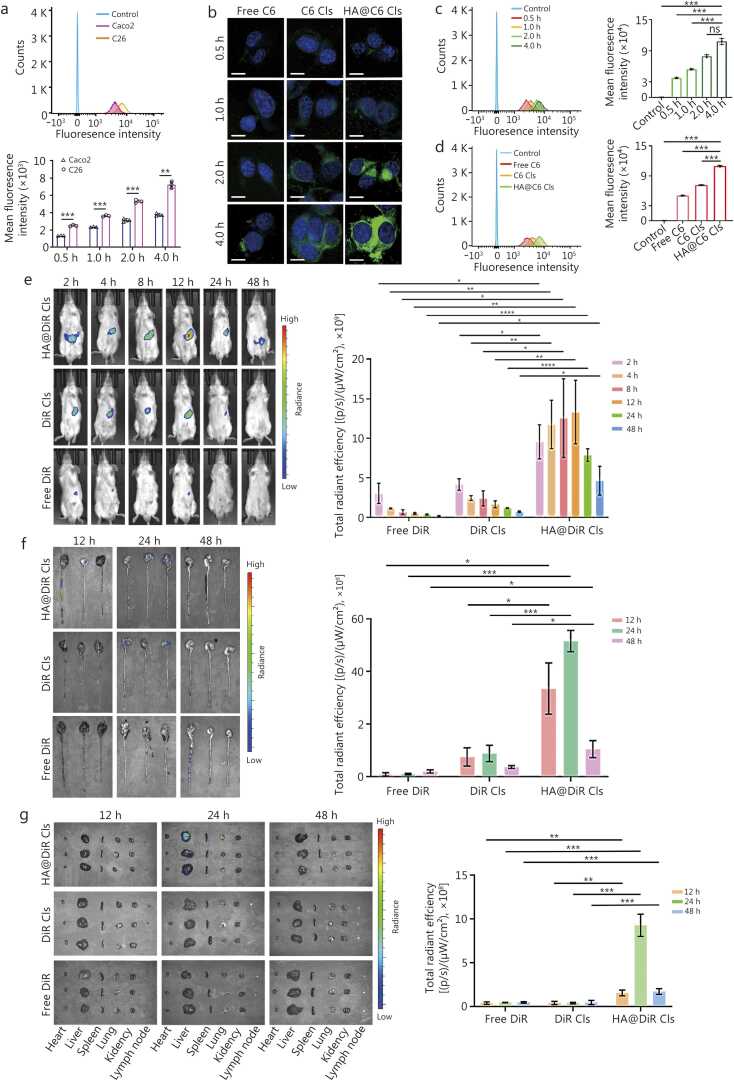
*In vitro* cellular uptake and *in vivo* biodistribution of nanotherapeutics. **a** Flow cytometry showing cellular uptake of HA C6 Cls in C26 and Caco2 cells. **b** CLSM images of cellular uptake of free C6, C6 Cls, and HA@C6 Cls at different timepoints in C26 cells. **c** Flow cytometry showing cellular uptake and mean fluorescence intensity analysis of HA@C6 Cls incubated with C26 cells for different times. **d** Flow cytometry showing cellular uptake and quantification analysis of free C6, C6 Cls, and HA@C6 Cls incubated with C26 cells for 4 h. **e***In vivo* imaging of mice and quantification at different timepoints after gavage administration. **f** Colorectum images and quantification of the tumor. **g** Ex vivo images of main organs and quantification in mesenteric lymph nodes. Data were presented as mean±SD, analyzed by one-way analysis of variance followed by Tukey’s post-test (a, c-g, *n=*3). ^⁎^*P*<0.05, ^⁎⁎^*P*<0.01, ^⁎⁎⁎^*P*<0.001, ^⁎⁎⁎⁎^*P*<0.0001, ns non-significant. CLSM. Confocal laser scanning microscopy; C6. coumarin 6; DiR. 1,1’-dioctadecyl-3,3,3’,3’-tetramethylindotricarbocyanine iodide; HA. Hyaluronic acid; Cls. Cationic liposomes; SD. Standard deviation.

The efficacy of drug delivery systems relies on efficient internalization by tumor cells *in vitro* and precise tumor tissue targeting *in vivo*. At the cellular level, NPs uptake was evaluated using two complementary approaches: qualitative confocal laser scanning microscopy (CLSM) and quantitative flow cytometry. To assess CD44 receptor-mediated targeting, we utilized HA@ Coumarin 6 (C6) Cls as fluorescent tracers, and uptake was compared between Caco2 and C26 cells. Quantitative analysis revealed markedly enhanced internalization of HA@C6 Cls in C26 cells, supporting CD44-specific targeting (Fig. 3**a, b**). Kinetic studies further demonstrated time-dependent cellular uptake of liposomes in C26 cells (Fig. 3**c**). Importantly, uptake of HA@C6 Cls was greater than that of C6 Cls and free C6 (Fig. 3**d**). Flow cytometry showed that HA@C6 Cls-mediated cellular uptake was 1.53-fold higher than C6 Cls and 2.16-fold higher than free C6 at 4.0 h (**Additional file 1:** Table S4**)**. These findings are consistent with CD44 receptor-mediated endocytosis as the dominant internalization pathway.

Based on these *in vitro* uptake results, we established an orthotopic CRC model in BALB/c mice to evaluate *in vivo* tumor targeting using real-time fluorescence imaging. The near-infrared tracer 1,1’-dioctadecyl-3,3,3’,3’-tetramethylindotricarbocyanine iodide (DiR) was encapsulated in HA@DiR Cls and DiR Cls for *in vivo* tracking. Fluorescence imaging revealed significantly enhanced tumor accumulation of HA@DiR Cls compared with that of DiR Cls and free DiR at every time point examined (Fig. 3**e**). HA@DiR Cls maintained prominent tumor-associated fluorescence for 48 h post administration, indicating extended tumor retention conferred by HA coating. By contrast, DiR Cls showed detectable but progressively diminishing tumor fluorescence from 2 to 24 h, consistent with passive accumulation via the enhanced permeability and retention effect, while free DiR was progressively decreasing within 12 h and showed minimal tumor accumulation. To further evaluate biodistribution, colorectum imaging was conducted at 12, 24, and 48 h post administration (Fig. 3**f**). It was confirmed that HA@DiR Cls exhibited greater tumor accumulation, which was 2.85-fold higher than that of DiR Cls at 48 h (**Additional file 1:** Table S5). Notably, HA@DiR Cls also showed substantial accumulation in major organs, particularly mesenteric lymph nodes (Fig. 3**g**), suggesting lymphatic trafficking capabilities.

To quantify intratumoral accumulation of the therapeutic cargo, Ag NPs and PTX levels were measured in tumor tissues after oral administration of HA@Ag NPs/PTX Cls at 5 pharmacokinetic time points selected based on maximal *in vivo* NPs fluorescence intensity. Significant tumor-specific accumulation of both agents was confirmed (**Additional file 1:** Fig. S2).

### 3.4 HA@Ag NPs/PTX Cls exhibit dual antibacterial activity and antitumor effects *in vitro*

**Fig. 4:**
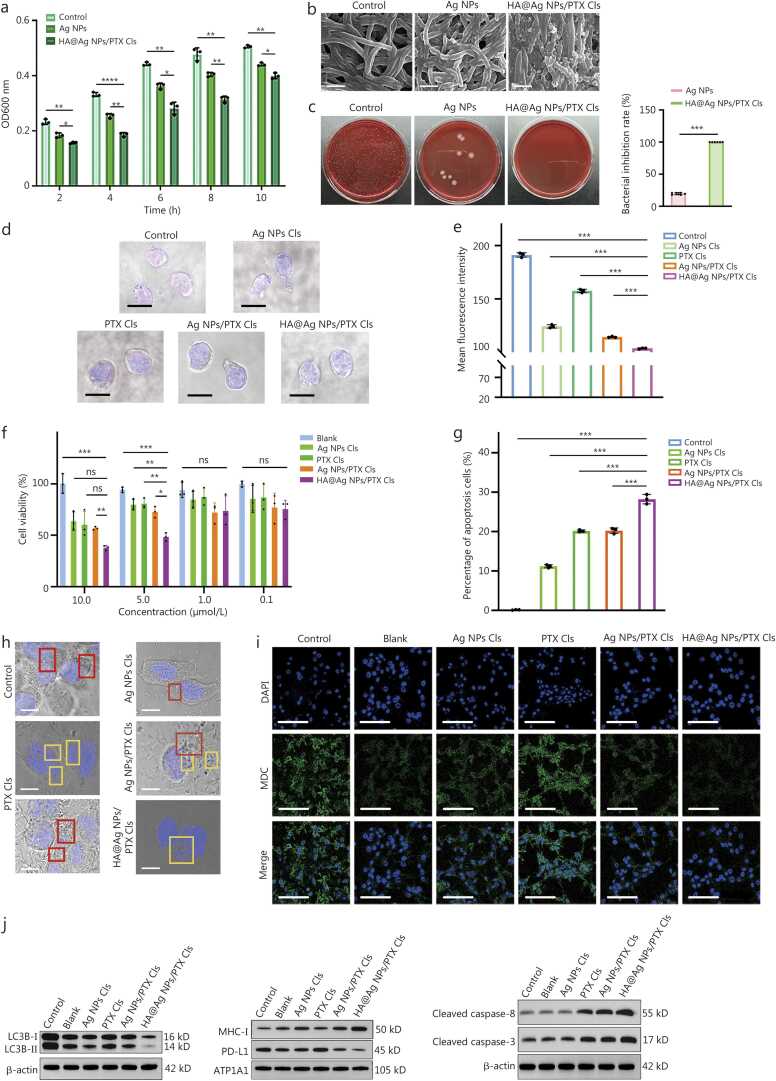
*In vitro* antibacterial and antitumor effects of the nanotherapeutics. **a** Growth inhibition curve of *F.n* under different treatments (*n*=3). **b** Surface morphology of *F.n* after co-culture with nanotherapeutics. Scale bar=1 μm. **c** Solid culture assays showing the antibacterial effects of co-culture with different nanotherapeutics (*n*=6). CLSM (**d**) and flow cytometric quantification (**e**) of infected tumor cells co-cultured with nanotherapeutics (*n*=3, DAPI: blue; EUB338: red). Scale bar=10 µm. **f** Viability of C26 cells following the indicated treatments (*n*=3). **g** Flow cytometry and quantitative analysis of apoptosis in cells stained with FITC and PI (*n*=3). **h** Representative photographs of the autophagosome and apoptotic vesicles following the indicated treatments (apoptotic vesicles in the yellow box and autophagosomes in the red box). Scale bar=10 µm. **i** CLSM images of autophagosomes. Scale bar=100 µm. **j** Western blotting analysis of proteins involved in the autophagy pathway, MHC-I, PD-L1, and proteins involved in the apoptosis pathway in C26 cells. Data were presented as mean±SD, analyzed by one-way analysis of variance followed by Tukey’s post-test (a, c, e-g). ^⁎^*P*<0.05, ^⁎⁎^*P*<0.01, ^⁎⁎⁎^*P*<0.001, ^⁎⁎⁎⁎^*P*<0.0001, ns non-significant. OD. Optical density; HA. Hyaluronic acid; Ag NPs. Silver nanoparticles; PTX. Paclitaxel; Cls. Cationic liposomes; DAPI. 4’;6-diamidino-2-phenylindole; MDC. Monodansylcadaverine; LC3B. Microtubule-associated protein 1 A/1B-light chain 3B; MHC-I. Major histocompatibility complex class I; PD-L1. Programmed death-ligand 1; ATP1A1. ATPase Na^+^/K^+^ transporting subunit alpha 1; CFU. Colony^-^forming units; SD. Standard deviation.

Based on the demonstrated tumor-targeting efficacy, we systematically evaluated the nanotherapy for antibacterial and antitumor effects *in vitro*. Liquid culture experiments revealed that *F.n* treated with HA@Ag NPs/PTX Cls exhibited significantly greater growth inhibition than that observed with Ag NPs alone or the control group (Fig. 4**a**). Scanning electron microscopy (SEM) analysis revealed striking morphological changes in treated *F.n*, bacteria in the control group maintained structural integrity with smooth membranes, whereas HA@Ag NPs/PTX Cls treatment results in severe membrane fragmentation (Fig. 4**b**). Consistent with these results, solid culture assays were showed 100% inhibition of colony formation by HA@Ag NPs/PTX Cls, compared with 19.6% inhibition by Ag NPs (Fig. 4**c**). Collectively, these results established HA@Ag NPs/PTX Cls as a potent bactericidal agent against *F.n*, likely mediated through membrane lysis mechanisms.

Next, we constructed an *F.n*-infected tumor cell model to evaluate the antibacterial efficacy of the different groups. *F.n* was stained with the EUB338 and visualized by confocal laser scanning microscopy (CLSM). HA@Ag NPs/PTX Cls treatment resulted in the weakest red fluorescence signal, indicating superior bacterial clearance compared with other groups (Fig. 4**d**). By contrast, Ag NPs Cls and PTX Cls exhibited the strongest fluorescence signals, indicating the highest residual bacterial loads. The Ag NPs/PTX Cls group exhibited higher fluorescence signal and higher bacterial loads compared to the HA@Ag NPs/PTX Cls group. Flow cytometry further confirmed that HA@Ag NPs/PTX Cls produce the lowest bacterial fluorescence intensity among all treatments (Fig. 4**e**). The superior antibacterial performance of HA@Ag NPs/PTX Cls may be attributable to the HA coating, which enhances bacterial membrane adhesion, together with the Cls surface charge that promotes electrostatic binding to negatively charged *F.n* membranes. This combined effect may contribute to the observed enhancement.

Antitumor effects were verified via cell counting kit-8 (CCK-8) assays, flow cytometric apoptosis analysis, tumor sphere formation, and cell scratch assays. Blank liposomes exhibited excellent biocompatibility, with no significant impact on tumor cell viability even at the highest tested concentration (Fig. 4**f**). By contrast, all drug-loaded formulations produced dose-dependent cytotoxicity. Among these, HA@Ag NPs/PTX Cls resulted in the lowest cell viability and showed significantly greater cytotoxicity than Ag NPs/PTX Cls, suggesting that the enhanced cellular uptake contributed to the increased antitumor effect. Conversely, Ag NPs Cls, and PTX Cls showed low cytotoxicity. To delineate the mechanism of cell death, tumor cells were co-treated with HA@Ag NPs/PTX Cls at the half-maximal inhibitory concentration along with the pan-caspase inhibitor Z-VAD-FMK, necroptosis inhibitor necrostatin-1, or ferroptosis inhibitor ferrostatin-1, following CCK-8 analysis. Z-VAD-FMK co-treatment increased cell viability from 57% to 84%, whereas necrostatin-1 and ferrostatin-1 had no significant effect, indicating that apoptosis was the predominant mode of cell death induced by HA@Ag NPs/PTX Cls (**Additional file 1:** Fig. S3a).

Then we quantified apoptosis in the *F.n*-infected tumor cell model using Annexin V-Fluorescein *in situ* hybridization (FITC)/propidium iodide (PI) staining. Consistent with biocompatibility assessments, blank liposomes induced negligible apoptosis. The apoptosis rates of PTX Cls and Ag NPs Cls were 20.16% and 10.90%, respectively, while the apoptosis rates of Ag NPs/PTX Cls is 20.23%. The highest apoptosis rate (28.20%) was observed in the HA@Ag NPs/PTX Cls group, demonstrating synergistic integration of PTX-induced apoptosis, Ag NPs-mediated autophagy regulation, and HA-driven CD44 targeting, collectively enhancing tumor cell death (Fig. 4**g; Additional file 1:** Fig. S3b).

Macroscopic tumor inhibition was further assessed using tumor sphere assays. PTX Cls and Ag NPs/PTX Cls exhibited a modest suppressive effect on tumor growth, whereas HA@Ag NPs/PTX Cls produced the smallest tumor spheres with looser internal structure and increased permeability, indicating reduced sphere pressure (**Additional file 1:** Fig. S3c). Scratch assays were used to evaluate cell migration. PTX Cls, Ag NPs/PTX Cls, and HA@Ag NPs/PTX Cls all significantly inhibited cell migration, with HA@Ag NPs/PTX Cls demonstrating wider scratch gaps across all time points, indicating a stronger antimigration effect (**Additional file 1:** Fig. S3d).

For comprehensive mechanistic exploration, we investigated the effects of the nanotherapy on autophagy and apoptosis in *F.n*-infected tumor cells. TEM revealed abundant autophagosomes (red box) in infected cells, consistent with *F.n*-induced autophagy (Fig. 4**h**). Notably, Ag NPs Cls reduced autophagosome numbers and increased cell permeability, while HA@Ag NPs/PTX Cls demonstrated near-complete autophagy suppression with abundant apoptotic vesicles (yellow box), demonstrating dual-pathway modulation. Autophagy visualization using monodansylcadaverine (MDC) staining (Fig. 4**i**) revealed intense autophagosome fluorescence in infected cells and minimal signal following HA@Ag NPs/PTX Cls treatment, confirming potent autophagy blockade.

Western blotting analysis of autophagy-related proteins revealed that *F.n* infection significantly upregulated LC3B-I/II conversion, consistent with autophagy activation [21]. While treatment with Ag NPs significantly reduced LC3B-I/II levels, HA@Ag NPs/PTX Cls produced the strongest inhibitory effect in C26 cells. Furthermore, HA@Ag NPs/PTX Cls significantly enhanced MHC-I expression and reduced PD-L1 levels, suggesting enhanced tumor immunogenicity. In the apoptotic pathway, HA@Ag NPs/PTX Cls induced the highest expression of cleaved caspase-3 and cleaved caspase-8 (Fig. 4**j; Additional file 1:** Fig. S3e), confirming concurrent autophagy suppression and apoptosis activation.

### 3.5 Targeting intratumoral bacteria potentiates antitumor efficacy and remodels the immunosuppressive TME *in vivo*

**Fig. 5:**
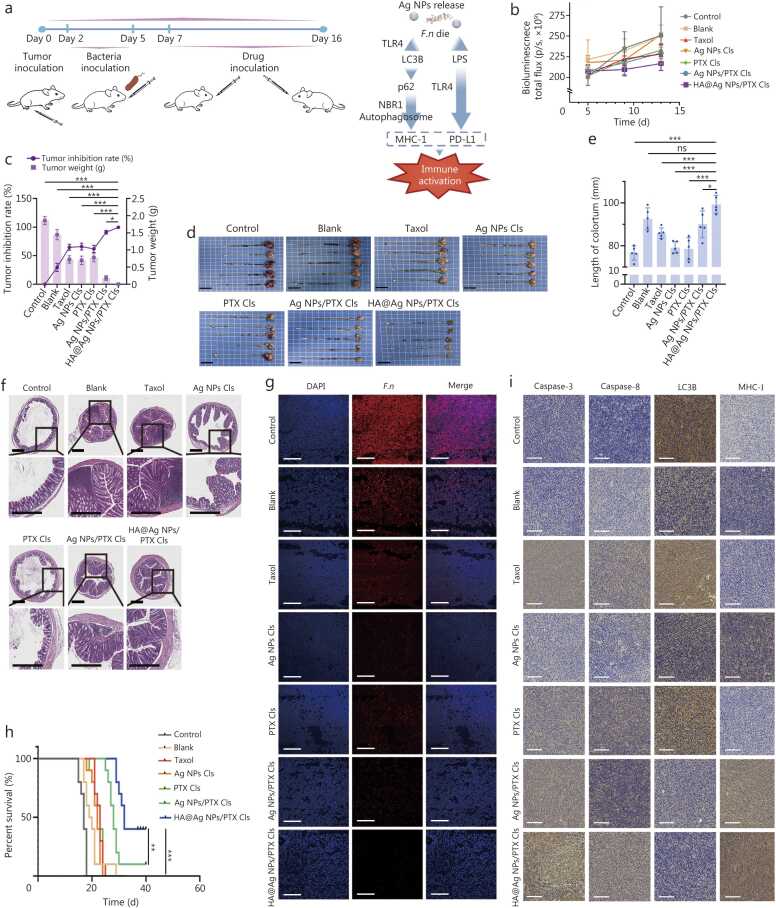
Nanotherapy-mediated delay in *F.n*-infected CRC progression *in situ.***a** Schematic of orthotopic CRC model construction and workflow with therapeutic mechanism. **b** Tumor fluorescence curve in mice undergoing nanotherapy (*n*=5). **c** Tumor inhibition rate and tumor weight at the end of treatment (*n*=5). **d** Representative photographs of C26 tumors and colorectum. **e** Colorectal length at the end of treatment. Scale bar=2 cm (*n*=5). **f** H&E staining of colorectal sections following the indicated treatments. Scale bar=500 μm. **g** CLSM images of fluorescence *in situ* hybridization to detect *F.n* infiltration in colorectal tumors. Scale bar=100 μm. **h** Overall survival rate of mice undergoing the indicated treatments (*n*=10). **i** IHC staining of autophagy and apoptosis pathway proteins and MHC-I in treated tumor tissues. Scale bar=100 nm. Data were presented as mean±SD. Data were analyzed by one-way ANOVA followed by Tukey’s post-test (b, c, e) and log-rank test (h). ^⁎^*P*<0.05, ^⁎⁎^*P*<0.01, ^⁎⁎⁎^*P*<0.001, ns non-significant. HA. Hyaluronic acid; Ag NPs. Silver nanoparticles; PTX. Paclitaxel; Cls. Cationic liposomes; DAPI. 4’,6-diamidino-2-phenylindole; *F.n*. *Fusobacterium nucleatum*; MHC-I. Major histocompatibility complex class I; PD-L1. Programmed death-ligand 1; TLR4. Toll-like receptor 4; LC3B. Microtubule-associated protein 1 A/1B-light chain 3B; LPS. Lipopolysaccharide; NBR1. Neighbor of *BRCA1* gene 1; SQSTM1. Sequestosome 1; SD. Standard deviation; CRC. Colorectal cancer; H&E. Hematoxylin and eosin; CLSM. Confocal laser scanning microscopy; IHC. Immunohistochemical.

By mimicking clinically relevant conditions, we assessed NPs efficacy in *F.n*-infected tumor-bearing BALB/c mice, which were grouped by their initial tumor fluorescence intensity and subsequently monitored every 4 d for changes in fluorescence intensity and body weight (Fig. 5**a**). Each of Taxol, Ag NPs Cls, PTX Cls, Ag NPs/PTX Cls, or HA@Ag NPs/PTX Cls all exhibited antitumor activity, with a significantly more pronounced effect observed in the HA@Ag NPs/PTX Cls group (Fig. 5**b**), which was accompanied by significant recovery of body weight (**Additional file 1:** Fig. S4a).

At the end of therapy (16 d), mice treated with HA@Ag NPs/PTX Cls achieved lower tumor weight, tumor volume, and a higher tumor inhibition rate compared with Ag NPs/PTX Cls, reflecting the critical role of HA-mediated targeting (Fig. 5**c; Additional file 1:** Fig. S4b). The lower antitumor efficacy of Taxol, Ag NPs Cls, and PTX Cls indicated that neither Ag NPs nor PTX alone can effectively inhibit tumor growth (Fig. 5**c, d**). Because colorectal shortening is strongly linked to dysfunction [22], colorectal length was measured to assess functional recovery. HA@Ag NPs/PTX Cls significantly alleviated shortening and improved colorectal function (Fig. 5**e**). Consistently, Ki-67 antigen expression in colonic tissue was markedly upregulated under HA@Ag NPs/PTX Cls compared with control, confirming enhanced mucosal proliferation (**Additional file 1:** Fig. S4c). Furthermore, H&E staining of colorectal tissues demonstrated significantly increased goblet cell numbers and crypt length, indicating that treatment with HA@Ag NPs/PTX Cls aided in the structural and functional restoration of the colorectum (Fig. 5**f**).

H&E and TUNEL staining demonstrated that HA@Ag NPs/PTX Cls induced tumor cellularity with loosened tissue architecture, and induced tumor cell apoptosis, confirming potent antitumor efficacy (**Additional file 1:** Fig. S4d). Meanwhile, EUB338 staining showed faint *F.n* infiltration in the HA@Ag NPs/PTX Cls group, verifying superior antibacterial efficacy (Fig. 5**g**).

Intratumoral bacteria are closely associated with tumor metastasis [23]. H&E staining revealed extensive metastatic nodules and widespread tumor cell distribution in the livers of control, blank, and PTX Cls groups, along with lung metastases and structural alterations in the control group. By contrast, no detectable metastases were observed in the HA@Ag NPs/PTX Cls group (**Additional file 1:** Fig. S4e), indicating a potent antimetastatic effect.

Median survival in mice was longest in the HA@Ag NPs/PTX Cls group (32 d), compared with control (17 d), blank (20 d), Taxol (23 d), Ag NPs Cls (22 d), PTX Cls (23 d), and Ag NPs/PTX Cls (28 d) (Fig. 5**h**).

Next, we further evaluated key indicators of the tumor immune microenvironment, including autophagy, apoptosis, and MHC-I expression. IHC analysis revealed that tumors treated with HA@Ag NPs/PTX Cls exhibited the highest levels of caspase-3 and caspase-8, consistent with Western blotting results in Fig. 4**j** above and indicating robust apoptosis induction (Fig. 5**i; Additional file 1:** Fig. S5). These tumors also showed the lowest LC3B expression and highest MHC-I expression, significantly differing from other groups, supporting a role in alleviating immunosuppression (Fig. 5**i; Additional file 1:** Fig. S5). Ag NPs/PTX Cls showed higher apoptosis-related protein expression than PTX Cls, potentially reflecting immune activation following bacterial clearance, which may boost TNF levels and exogenous apoptosis [24]. Collectively, these findings demonstrated that *F.n* elimination can activate the immune microenvironment and promote tumor cell apoptosis *in vivo*.

Immune cells were analyzed in the tumor, spleen, and mesenteric lymph nodes using flow cytometry with parallel cytokine measurements by ELISA, for comprehensive immune response assessment. Compared with other groups, HA@Ag NPs/PTX Cls significantly increased central memory T cells (T_CM_) in the spleen and mature dendritic cells (mDCs) in the mesenteric lymph nodes, suggesting improved antigen presentation and long-term immune activation. Additionally, HA@Ag NPs/PTX Cls significantly increased cluster of differentiation 8 positive (CD8^+^) T-cell infiltration in tumor tissues compared with other groups, thereby enhancing immune-mediated tumor clearance (**Additional file 1:** Fig. S6). Evaluation of tumor cytokines revealed that HA@Ag NPs/PTX Cls significantly upregulated IFN-α and IFN-γ, and downregulated PD-L1, indicating immune microenvironment activation, and markedly increased TNF-α, which induced tumor cell apoptosis via the extrinsic pathway (**Additional file 1:** Fig. S7).

### 3.6 Eliminating intratumoral bacteria prevents tumor recurrence by stimulating long-term antitumor immunity

**Fig. 6:**
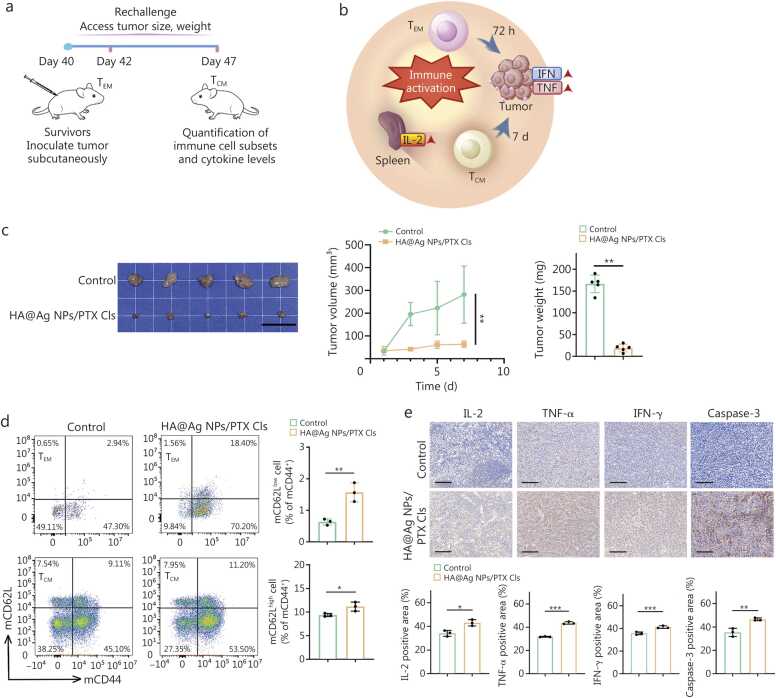
Rechallenge experiment of long-term survivors of orthotopic tumors. **a** Schematic of the rechallenge model. **b** Immune cell recruitment process. **c** Tumor volume during the experiment, tumor photographs, and tumor weight at the end of the experiment. Scale bar=2 cm (*n*=5). **d** Flow cytometry analysis of T_EM_ among CD8^+^ T cells in tumors and T_CM_ among CD8^+^ T cells in the spleen following rechallenge (*n*=3). **e** IHC staining of IL-2 in the spleen, TNF-α, IFN-γ, and caspase-3 in tumor tissues with quantitative evaluation (*n*=3). Scale bar=100 μm. Data were presented as mean ± SD, analyzed by Student’s *t*-test (**c**-**e**). ^⁎^*P*<0.05, ^⁎⁎^*P*<0.01, ^⁎⁎⁎^*P*<0.001. HA. Hyaluronic acid; Ag NPs. Silver nanoparticles; PTX. Paclitaxel; Cls. Cationic liposomes; T_CM_. Central memory T cells; T_EM_. Effector memory T cells; TNF-α. Tumor necrosis factor-α; IL-2. Interleukin-2; IFN. Interferon; SD. Standard deviation; CD62L. Cluster of differentiation 62L; CD44. Cluster of differentiation 44; IHC. Immunohistochemical.

To further investigate *F.n*-mediated long-term immunity, we established a rechallenge model using long-term survivors from the orthotopic tumor model, based on T_CM_ upregulation in the spleen, alongside PBS control-treated mice. Secondary tumors were inoculated near the inguinal lymph nodes to evaluate the long-term immunological memory following HA@ Ag NPs/PTX Cls treatment (Fig. 6**a**).

Memory T cells were classified into effector memory T cells (T_EM_), T_CM_, and tissue-resident memory T cells (T_RM_) [15], [25]. We focused on systemic rather than colorectal-restricted effects by comparing tumor-infiltrating T_EM_ and splenic T_CM_. Upon rechallenge, T_EM_ infiltrated the TME within 72 h, mediating tumor cell elimination and antigen release [16]. To examine this early response, tumors were collected 48 h after the secondary inoculation for T_EM_ quantification. Simultaneously, T_CM_ in the spleen underwent clonal expansion, differentiated into effector T cells, and migrated to tumors, with spleens analyzed at day 7 to assess systemic memory responses (Fig. 6**b**) [25]. Compared with the control group, survivors demonstrated markedly reduced tumor volumes and tumor weights (Fig. 6**c**) and markedly increased frequencies of tumor-infiltrating T_EM_ and splenic T_CM_ populations (Fig. 6**d**).

We conducted a comparative cytokine analysis for further evaluation of immune responses. Given that IL-2 promotes T_CM_ expansion, while T_EM_-associated TNF-α and IFN-γ mediate tumor cell death, we quantified IL-2 levels in the spleen and TNF-α and IFN-γ levels in tumors [15], [16]. Survivors showed high splenic expression of IL-2 and elevated tumoral expression of TNF-α and IFN-γ. Furthermore, caspase-3 assessment revealed enhanced tumor apoptosis in survivors compared with the control group (Fig. 6**e**). Collectively, *F.n* elimination created a favorable and durable immunomodulatory microenvironment that effectively prevented tumor recurrence.

### 3.7 Biosafety evaluation of HA@Ag NPs/PTX Cls

First, we assessed intestinal biosafety by comparing HA@Ag NPs/PTX Cls cytotoxicity in C26 and Caco2 cells. C26 cells exhibited stronger activation of cleaved caspase-3 than Caco2 cells, confirming HA-targeted specificity (**Additional file 1:** Fig. S8a). Next, we conducted a comprehensive toxicological evaluation. To assess acute toxicity, mice were administered therapeutic-dose equivalents, with no significant differences in body weight observed between the HA@Ag NPs/PTX Cls-treated and blank groups (vehicle control) (**Additional file 1:** Fig. S8b). After 15 d of continuous administration, mice were euthanized for serum and tissue analyses. Serum biochemical markers (CRE, BUN, AST, and ALT) showed no hepatotoxicity or nephrotoxicity (**Additional file 1:** Fig. S8c), and H&E staining confirmed good biocompatibility of HA@Ag NPs/PTX Cls (**Additional file 1:** Fig. S8d). Long-term toxicity was assessed at 1×, 5×, and 10× therapeutic doses for 28 d. Only mild intestinal crypt atrophy was observed at the 10× dose, with no other significant effects in other tissues at any dose, demonstrating a favorable safety profile for HA@Ag NPs/PTX Cls at therapeutic doses (**Additional file 1:** Fig. S8e).

### 3.8 Post-treatment alterations in intratumoral flora

To improve the resolution of conventional 16S rDNA gene sequencing, we employed 5 R 16S sequencing, a novel strategy widely adopted in intratumoral bacterial studies for more accurate species-level classification [26], [27]. Analysis of alpha-diversity indices (observed species, Chao1, Shannon, and Simpson) in tumor samples revealed lower richness in tumor-bearing *F.n*-infected mice (control group) than in tumor-bearing *F.n*-uninfected mice (blank group), confirming successful infection modeling. Notably, bacterial richness further increased in tumors from the HA@Ag NPs/PTX Cls group compared with that in the Taxol and control groups (**Additional file 1:** Fig. S9a). Beta-diversity analysis via PCoA indicated overall shifts in the tumoral bacterial community, with HA@Ag NPs/PTX Cls inducing significant structural changes relative to controls, suggesting suppression of harmful bacteria and potential enrichment of beneficial species (**Additional file 1:** Fig. S9b).

From the phylum to genus levels, dominant flora markedly differed between the control and HA@Ag NPs/PTX Cls groups. *F.n* dominated in the controls, but showed reduced abundance following treatment, confirming effective bacterial modulation (**Additional file 1:** Fig. S10). Concurrently, the type and abundance of beneficial bacteria, such as *Clostridium* spp., increased in the HA@Ag NPs/PTX Cls group, potentially contributing to tumor growth inhibition [5]. An evolutionary plot of intratumoral bacteria further illustrated distinct compositional structures between HA@Ag NPs/PTX Cls-treated tumors and other groups (**Additional file 1:** Fig. S11).

## 4 Discussion

Intratumoral bacteria can influence tumor progression, with *F.n* promoting tumor growth by modulating the tumor immune microenvironment [28]. Our reanalysis of clinical samples further confirmed that *F.n* enrichment in tumor tissues modulates the TME. Conversely, intratumoral probiotics can decelerate tumor growth through multiple mechanisms [4], highlighting the critical role of bacterial balance in tumor progression. Such imbalances likely contribute to *F.n*’s modulation of the tumor immune microenvironment.

We verified that *F.n* subverts antitumor immunity via dual mechanisms involving MHC-I degradation and PD-L1 upregulation. These alterations induced immune suppression and autophagy dysregulation, which act as major obstacles to mounting an effective antitumor immune response (Fig. 1**j, l**) [17], [29]. Building upon clinical data reanalysis and mechanistic validation, we engineered HA@Ag NPs/PTX Cls to remodel the TME. NPs were synthesized via film dispersion and electrostatically coated with HA, yielding high drug-loading efficiency, uniform particle size, gastrointestinal stability, and CD44-targeting specificity (Figs. 2 **and** 7**a**). These data collectively demonstrated that HA-coated liposomes significantly enhanced tumor-targeting delivery and facilitated lymphatic distribution, highlighting the dual potential for improved drug bioavailability and antimetastatic effects in CRC.

**Fig. 7:**
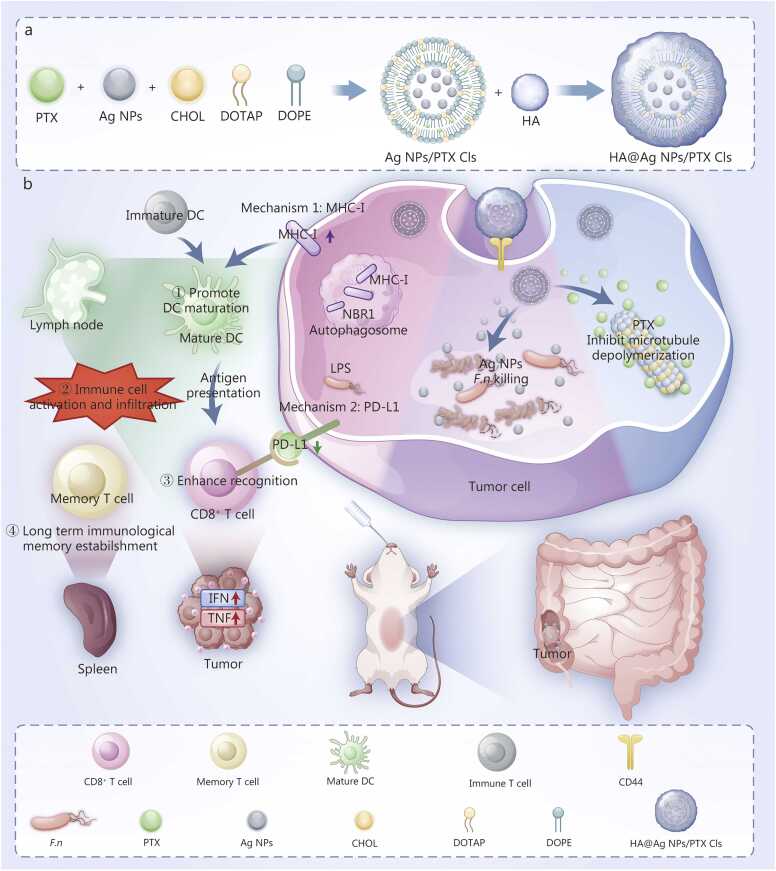
Construction and mechanistic investigation of targeted nanotherapy for CRC. **a** Preparation of HA@Ag NPs/PTX Cls. **b** Antitumor mechanisms aimed at modulating the tumor microenvironment and enhancing the apoptosis pathway. CRC. Colorectal Cancer; HA. Hyaluronic acid; Ag NPs. Silver nanoparticles; PTX. Paclitaxel; Cls. Cationic liposomes; DC. Dendritic cell; DOPE. 1;2-dioleoyl-SN-glycero-3-phosphoethanolamine; DOTAP. 2-dioleoyl-3-trimethylammonium propane; CHOL. Cholesterol; MHC-I. Major histocompatibility complex class I; PD-L1. Programmed death-ligand 1; LPS. Lipopolysaccharide; NBR1. Neighbor of *BRCA1* gene 1; CD44. Cluster of differentiation 44; *F.n*. *Fusobacterium nucleatum*; TNF-α. Tumor necrosis factor-α; IFN. Interferon.

These HA@Ag NPs/PTX Cls effectively eliminated *F.n* and inhibited tumor growth both *in vitro* and *in vivo* (Fig. 4, Fig. 5), validating the approach of restoring the tumor immune microenvironment through targeted *F.n* eradication. Our findings further revealed that HA@Ag NPs/PTX Cls suppress autophagosome formation [21] and elevate MHC-I expression, reflecting inhibition of autophagy activation. Meanwhile, this treatment markedly reduced *F.n* LPS-induced PD-L1 upregulation (Figs. 4**j and** 7**b**), indicating relief of tumor immune suppression. By simultaneously enhancing MHC-I expression and lowering PD-L1, HA@Ag NPs/PTX Cls promote immune cell recruitment and tumor recognition, while integrating PTX-induced apoptosis with immune microenvironment activation. This dual mechanism underlies the formulation’s antitumor efficacy and its ability to augment chemotherapy.

Consistent with *in vitro* results, our *in vivo* experiments showed dysregulated autophagy in tumor cells. Apoptosis-related protein expression was higher in the Ag NPs/PTX Cls group than in the PTX Cls group, likely reflecting immune-mediated TNF upregulation and enhancement of exogenous apoptosis following *F.n* eradication by Ag NPs [24]. These findings indicate that eliminating intratumoral bacteria can both reverse the immunosuppressive TME and promote tumor cell apoptosis. HA@Ag NPs/PTX Cls triggered robust immune activation, as evidenced by the enrichment of immune cells and elevated levels of MHC-I, IL-2, TNF, and IFN *in vivo*. These findings indicate that the treatment promotes DC maturation [30], enhances antigen presentation, strengthens cytotoxic immune cell responses, and induces durable immunological memory. Through these multimodal mechanisms, HA@Ag NPs/PTX Cls effectively prevented tumor recurrence and remodeled the TME (Fig. 6).

Finally, 5 R 16S rDNA sequencing demonstrated that HA@Ag NPs/PTX Cls effectively eliminated *F.n*, increased bacterial richness, and induced structural changes in the intratumoral bacterial community, including enrichment of probiotic species that may affect tumor progression. Although different tumor types harbor distinct microbial communities, tumor-associated bacteria frequently employ similar mechanisms to facilitate cancer progression. As demonstrated here, *F.n* promotes malignancy by activating autophagy, downregulating MHC-I, and upregulating PD-L1, thereby reshaping the tumor immune microenvironment. Accordingly, this nanotherapy, designed to eliminate *F.n* and reverse immunosuppression, may have broad applicability within the context of CRC treatment.

This study has several limitations. First, the absence of a combination therapy group incorporating immune checkpoint inhibitors limits direct clinical benchmarking against standard regimens. Second, although immune microenvironment analysis included mature DCs, CD8^+^ T cells, T_CM_, and T_EM_, it did not encompass other relevant immune populations (e.g., regulatory T cells, natural killer cells, myeloid-derived suppressor cells). Third, as metal-based nanomaterials, Ag NPs require thorough evaluation of long-term systemic toxicity and clinical translatability. Finally, although HA@Ag NPs/PTX Cls were taken up by tumor cells via HA-mediated tumor cell targeting and effectively eliminated intratumoral *F.n*, their broad-spectrum antibacterial activity may also impact non-target bacterial populations. Restoration of the bacterial community and repopulation of probiotics is likely to require a temporal progression.

## 5 Conclusions

Building upon existing clinical data from CRC patients, we developed a dual-functional nanotherapy that synergistically eliminates tumor-associated bacteria while enhancing chemotherapeutic efficacy. This platform combines bacterial clearance, which reverses bacteria-mediated immunosuppression, with induction of tumor cell apoptosis, demonstrating potent antitumor activity in CRC. Our approach establishes a novel strategy for intratumoral bacteria-regulated nanotherapy, which may provide insights for future clinical translation.

## Abbreviations

Ag NPs: Silver nanoparticles

ALT: Alanine aminotransferase

AST: Aspartate aminotransferase

BUN: Blood urea nitrogen

C6: Coumarin 6

CCK-8: Cell counting kit-8

CD8: Cluster of differentiation 8

CD44: Cluster of differentiation 44

CFU: Colony-forming units

Cls: Cationic liposomes

CRC: Colorectal cancer

CRE: Creatinine

DC: Dendritic cell

DiR: 1,1’-dioctadecyl-3,3,3’,3’-tetramethylindotricarbocyanine iodide

EUB338: Eubacteria probe 338

EE: Encapsulation efficiency

ELISA: Enzyme-linked immunosorbent assay

*F.n*: Fusobacterium nucleatum

HA: Hyaluronic acid

H&E: Hematoxylin and eosin

IFN: Interferon

IHC: Immunohistochemical

IL-2: Interleukin-2

LC3B: Microtubule-associated protein 1 A/1B-light chain 3B

LPS: Lipopolysaccharide

MHC-I: Major histocompatibility complex class I

NBR1: Neighbor of *BRCA1* gene 1

PBS: Phosphate-buffered saline

PCR: Polymerase chain reaction

PDI: Polydispersity index

PD-L1: Programmed death-ligand 1

PTX: Paclitaxel

siRNA: Small interfering RNA

T_CM_: Central memory T cells

T_EM_: Effector memory T cells

TEM: Transmission electron microscopy

TLR4: Toll-like receptor 4

TME: Tumor microenvironment

TNF-α: Tumor necrosis factor-α

T_RM_: Tissue-resident memory T cells

## Ethics approval and consent to participate

Clinical data obtained from the National Center for Biotechnology Information (NCBI) public repository (https://www.ncbi.nlm.nih.gov/; Accession: PRJNA811533) were regrouped and reanalyzed. Animal experiments were performed under the guidelines of the Laboratory Animal Ethics Committee of the Institute of Materia Medica, Chinese Academy of Medical Sciences, and Peking Union Medical College (IMM-N-25-0338 and 00004435).

## Funding

This work was supported by the CAMS Innovation Fund for Medical Sciences (CIFMS) (2021-I2M-1-026).

## Data Availability

The authors confirm that the data supporting the findings of this study are available within the article and its Additional file 1.

## Footnotes

Appendix A Supplementary data associated with this article can be found in the online version at doi:10.1016/j.mmr.2026.100036.
