# Supplementary material for: Targeting intratumoral bacteria reverses MHC-I suppression and enhances nano-based chemoimmunotherapy in colorectal cancer
Source: Mil Med Res. 2026 May 7;13(1):100036. doi: 10.1016/j.mmr.2026.100036 (PMC13185994; doi:10.1016/j.mmr.2026.100036)
Supplement: Supplementary file 1 — Supplementary material [file mmc1.pdf]

Methods

Materials and agents

The methyl sulfate salts of 1,2-dioleoyl-3-trimethylammonium propane (DOTAP) and 1,2-dioleoyl-SN-glycero-3-phosphoethanolamine (DOPE) were purchased from InnoChem Science & Technology Co., Ltd. (Beijing, China). Cholesterol was obtained from Sinopharm Chemical Reagent Co., Ltd. (Shanghai, China). Silver nanoparticles (Ag NPs) were purchased from XFNANO Materials Technology Co., Ltd. (Nanjing, China). The Cell Counting Kit-8 (CCK-8) was from APE×BIO Technology LLC (Shanghai, China).

Defibrillated sheep blood, 4',6-diamidino-2-phenylindole (DAPI), fluorescein isothiocyanate (FITC)-annexin V/propidium iodide (PI) apoptosis detection kit, enzyme-linked immunosorbent assay (ELISA) kits for interferon-α (IFN-α), IFN-γ, and tumor necrosis factor-α (TNF-α), and the mycoplasma detection kit were obtained from Solarbio Science & Technology Co., Ltd. (Beijing, China). Qubit bacterial DNA extraction Kit was purchased from Thermo Fisher Scientific Invitrogen (Waltham, MA 02451, USA). Coumarin 6 (C6) was purchased from Sigma-Aldrich (St. Louis, MO, USA).

Fusobacterium nucleatum (*F.n*) was purchased from Beijing BioDee Biotechnology Co., Ltd. (Beijing, China), and the eubacteria probe 338 (EUB338) was from Shanghai Weiji Biotechnology Co., Ltd. (Shanghai, China). Anti-cluster of differentiation 8 (CD8) antibody was obtained from Selleck Chemicals LLC (Houston, TX, USA). Anti-major histocompatibility complex class I (MHC-I), anti-caspase-3, anti-caspase-8, anti-sequestosome 1 (p62), anti-microtubule-associated protein 1A/1B-light chain 3B (LC3B), anti-Toll-like receptor 4 (TLR4), and anti-neighbor of BRCA1 gene 1 (NBR1) antibodies were obtained from Cell Signaling Technology Inc. (Danvers, MA, USA). All antibodies used for immunocytological experiments were purchased from BioLegend (San Diego, CA, USA).

Liposome composition

DOTAP was a cationic quaternary ammonium lipid. Its hydrophobic oleoyl chains are embedded into the bilayer, while the positively charged trimethylammonium headgroup faces outward, thereby stabilizing the structure via electrostatic interactions. DOPE exhibited a conical molecular geometry that promoted non-bilayer phase formation and enhanced membrane fusion. Cholesterol incorporated into phospholipid bilayers via its steroidal ring, reducing membrane fluidity through hydrophobic

interactions and enhancing liposomal stability.

Cationic liposomes (Cls) characterization

The size and zeta potential of Cls were measured by dynamic light scattering (DLS) using a Zetasizer Nano ZS90 (Malvern Instruments Ltd., UK).

The encapsulation efficiency (EE) of paclitaxel (PTX) was determined using high-performance liquid chromatography (HPLC) and calculated as follows: EE%=(weight of loaded drug)/(total weight of drug)×100%. HPLC analysis of PTX was performed on a C18 column using acetonitrile-water as the mobile phase (flow rate: 1.0 ml/min; column temperature: 30 °C; detection wavelength: 227 nm; injection volume: 20 µl; time: 20 min (Agilent 1260; Agilent Technologies Inc., USA).

Ag NPs were quantified by measuring their characteristic absorption peak at 410 nm using ultraviolet-visible (UV-vis) spectroscopy (T6; Beijing Persee General Instrument Co., Ltd., China).

Hyaluronic acid (HA) coating efficiency was evaluated following electrostatic adsorption onto Cls and calculated as follows: coating efficiency (%)=[1–(free HA)/(total HA)]×100%. Free HA was separated by ultrafiltration [molecular weight cut-off (MWCO): 100 kD] and quantified using ELISA based on competitive binding to recombinant HA polysaccharide pre-coated on microplates. After incubation, biotinylated recombinant HA polysaccharide and horseradish peroxidase-conjugated streptavidin were sequentially added, and color development was achieved using 3,3’,5,5’-tetramethylbenzidine (TMB) substrate. Absorbance was measured at 450 nm (T6; BioTek Inc., USA).

Electron microscopy of liposomes

Liposome morphology was examined using transmission electron microscopy (TEM) and scanning electron microscopy (SEM). For TEM analysis, liposome suspensions (0.1 mg/ml) were loaded onto carbon-coated 300-mesh copper grids for 60 s, negatively stained with 2% phosphotungstic acid for 30 s, washed with ultrapure water, and air-dried at 25 °C for 12 h prior to imaging (HT7800; Hitachi, Ltd., Japan) [1]. For SEM analysis, liposome suspensions were ultrasonicated, deposited onto conductive adhesive substrates, and air-dried for 24 h before imaging (Apero; Thermo Fisher Scientific Inc., USA) [2].

***In vitro* drug release**

NPs (1 ml) were loaded into a dialysis bag (MWCO: 10 kD) and dialyzed against 20 ml of release medium with constant shaking at 37 °C. The dialysis bag was sequentially incubated in artificial gastric

fluid for 2 h, artificial small intestinal fluid for 2–6 h, and artificial colonic fluid for 6–18 h. At each time point, samples of the release medium were collected and immediately replaced with an equal volume of fresh medium. Cumulative release was calculated, and the *in vitro* release profile was plotted.

***F.n* culture**

F.n was cultured in thioglycolate broth at 37 °C for 48 h under anaerobic conditions. Bacterial cells in the logarithmic growth phase were harvested by centrifugation at 6000× *g* for 10 min, washed twice with phosphate-buffered saline (PBS), and resuspended in PBS for subsequent experiments.

For solid culture, *F.n* was streaked onto Columbia blood agar plates and incubated at 37 °C for 72 h under anaerobic conditions. Distinct single colonies were selected for subculture [3].

***In vitro* cellular uptake**

F.n-infected tumor cells were incubated with C6-loaded liposomes for 4 h under standard culture conditions. C6-loaded Cls (C6 Cls) and HA@C6 Cls were prepared following the same protocol as described for HA@Ag NPs/PTX Cls, with a final C6 concentration of 1 μg/ml. After incubation, cells were washed with ice-cold PBS, fixed with 4% paraformaldehyde for 15 min at room temperature, and counterstained with DAPI (excitation: 358 nm; emission: 461 nm). C6 fluorescence was detected at excitation 466 nm/emission 504 nm. Cellular internalization was visualized using confocal laser scanning microscopy (CLSM; TCS SP2; Leica Microsystems GmbH, Germany).

For quantitative analysis, cells were trypsinized and subjected to flow cytometry (Verse; Becton, Dickinson and Company, USA), with fluorescence intensity and uptake percentage calculated using FlowJo software. To assess HA targeting specificity, NPs uptake was compared between Caco2 and C26 cells at 0.5, 1.0, 2.0, and 4.0 h using flow cytometry.

***In vivo* imaging and biodistribution analysis**

In vivo liposome distribution was tracked using an *in vivo* imaging system (IVIS, Spectrum CT; Caliper Life Sciences Inc., USA). Tumor-bearing mice were assigned to groups based on fluorescence intensity. Following oral administration of free 1,1’-dioctadecyl-3,3,3’,3’-tetramethylindotricarbocyanine iodide (DiR), DiR-loaded Cls (DiR Cls), or HA@DiR Cls, whole-body imaging of mice was performed at 2, 4, 8, 12, 24, and 48 h using 640 nm excitation and 680 nm emission wavelengths.

For *ex vivo* biodistribution analysis, mice were euthanized at 12, 24, and 48 h post administration. Tumors and major organs were excised immediately and subjected to fluorescence imaging using the

IVIS system. DiR Cls and HA@DiR Cls were prepared following the same protocol as previously described for HA@Ag NPs/PTX Cls, with the DiR dosage standardized to 1 mg/kg body weight.

***In vitro* antibacterial experiments**

Bacterial growth was quantified using absorbance at 600 nm (UV-vis). Cells from each drug treatment group were co-incubated with log-phase bacteria in liquid medium, and bacterial density was recorded at 2, 4, 6, 8, and 10 h. For colony-forming units (CFU) determination, a 50 μl sample of each culture was diluted 100-fold, spread onto blood agar plates, and incubated prior to counting. To examine bacterial morphology, liquid cultures were centrifuged, and bacterial pellets were fixed with 2.5% glutaraldehyde, dehydrated through a graded ethanol series, and observed using SEM.

***In vitro* antibacterial efficacy in infected tumor cells**

Antibacterial effects of NPs were evaluated in C26 cells infected with *F.n*-infected cells were co-incubated with different NPs for 4 h, then fixed. Bacterial cells were fluorescently labeled using EUB338 and DAPI for CLSM (TCS SP8X; Leica Microsystems GmbH, Germany) imaging (excitation: 586 nm; emission: 605 nm).

For bacterial quantification, infected cells were enzymatically dissociated and analyzed by flow cytometry (Verse; Becton, Dickinson and Company, USA).

Tumor cell viability assay

C26 cells were seeded in 96-well plates (5000 cells/well) and cultured in complete medium for 24 h. Infected cell models were established, and cells were co-incubated with different drugs for 4 h, then maintained in serum-supplemented medium. After 1 h incubation with CCK-8 reagent (0.5 mg/ml), absorbance was measured at 450 nm using a microplate reader.

Tumor cells were treated with HA@Ag NPs/PTX Cls at their half-maximal inhibitory concentration (IC_{50}) together with the pan-caspase inhibitor Z-VAD-FMK (20 μmol/L), the necroptosis inhibitor necrostatin-1 (10 μmol/L), and the ferroptosis inhibitor ferrostatin-1 (1 μmol/L). Cell viability was subsequently analyzed using the CCK-8 assay (Synergy H1; BioTek Inc., USA).

Tumor cell apoptosis assay

C26 cells were seeded in 12-well plates at 3×10^5 cells/well and cultured in complete medium for 24 h. Infected cell models were established, and cells were treated with the indicated drugs for 4 h. After

washing with PBS, cells were further cultured in complete medium for 24 h, trypsinized, and dual-stained with annexin V-FITC and PI. Apoptosis was analyzed by flow cytometry (Verse; Becton, Dickinson and Company, USA), with excitation at 488 nm and emission captured at 530/30 nm for FITC and 610/20 nm for PI.

Tumor sphere experiments

Cells were seeded in 96-well plates pre-coated with agarose gel at a density of 5000 cells/well. When tumor spheres reached 800 μm in diameter, treatment with the indicated drugs was initiated. The tumor spheres were photographed and documented on days 1, 3, and 5.

Tumor cell migration assay

C26 cells were seeded in 12-well plates at 3×10^5 cells/well and cultured for 24 h. A scratch was created in the monolayer using a 200 μl pipette tip, and cells were subsequently treated with NPs. Migration into the wound area was observed at 0, 12, 24, and 36 h post treatment.

Pharmacokinetic analysis in mice

At designated time points, tumor tissues were collected, weighed, and 200 mg aliquots were homogenized for quantification of Ag NPs and PTX. For Ag NPs quantification, tissue samples were digested with 20 μg/ml proteinase K at 55 °C for 4 h, and the reaction was terminated with 1 mmol/L phenylmethylsulfonyl fluoride. Proteins were extracted using a chloroform-isopropanol mixture (3:1 v/v) followed by centrifugation, and the aqueous phase was collected. Ag NPs concentrations were determined by measuring the characteristic absorption peak at 410 nm. For PTX quantification, tissue homogenates were diluted with isotonic sodium chloride solution, and proteins were precipitated with methanol containing 100 ng/ml wendoling (internal standard). After centrifugation (15,000× *g*, 10 min, 4 °C), the supernatant was analyzed by liquid chromatography on a Zorbax SB-C18 column at 30 °C. The mobile phase consisted of 0.1% formic acid and 10 mmol/L ammonium acetate in water-acetonitrile (40:60, v/v), with a flow rate of 0.25 ml/min, an injection volume of 5 μl, and a total run time of 6 min.

Mass spectrometric detection was conducted in positive electrospray ionization mode using selected reaction monitoring transitions: PTX, m/z 876.5 → 308.0; wendoling, *m/z* 457.3 → 188.1.

Autophagic vesicle observation

C26 cells were seeded in 12-well plates at a density of 3×10^5 cells /well and cultured for 24 h. *F.n*-infected tumor cells were established, co-incubated with NPs for 4 h, then washed and further incubated for 24 h. Cells were then fixed, and nuclei and autophagosomes were stained with DAPI and monodansylcadaverine (MDC), respectively. MDC-labeled autophagosomes were visualized using CLSM (excitation wavelength: 380 nm; emission wavelength: 525 nm).

Western blotting of autophagy and apoptosis pathway proteins

Western blotting was used to quantify proteins associated with autophagy and apoptosis. C26 cells were seeded in 6-well plates at a density of 1×10^6 cells/well and cultured for 24 h. Where indicated, cells were transfected with gene-specific small interfering RNAs (siRNAs) for 48 h to knock down expression of the target protein. After establishing the *F.n*-infected tumor cell model, cells were treated with NPs for 24 h. Cells were then trypsinized and lysed in buffer supplemented with protease and phosphatase inhibitors. Protein concentrations were determined using a bicinchoninic acid assay. Equal amounts of protein were mixed with sodium dodecyl sulfate (SDS) loading buffer, denatured by boiling, separated by SDS-polyacrylamide gel electrophoresis, and transferred onto a nitrocellulose membrane. The membrane was blocked with milk, incubated with primary antibody for 12 h at 4 °C, washed, and probed with the corresponding secondary antibody. Protein bands were visualized using an enhanced chemiluminescence (ECL; Bio-Plex 200; Bio-Rad, USA) prime reagent [4-6].

IHC analysis of autophagy and apoptosis pathway proteins *in vivo*

Paraffin-embedded tumor sections were deparaffinized, subjected to antigen retrieval, and blocked. Sections were incubated with primary antibody, followed by horseradish peroxidase-conjugated secondary antibody, and immunoreactivity was visualized using 3,3'-diaminobenzidine (DAB) as the chromogen.

Flow cytometry analysis of the immunotherapeutic mechanism

Tumors, mesenteric lymph nodes, and spleens were collected for immune cell profiling using flow cytometry. Infiltrating central memory T cells (T_{CM}) in the spleen were stained with FITC-conjugated anti-mouse cluster of differentiation 3 (CD3), phycoerythrin (PE)-conjugated anti-mouse cluster of differentiation 44 (CD44), and allophycocyanin (APC)-conjugated anti-mouse l-selectin (CD62L). Mature dendritic cell (mDCs) in mesenteric lymph nodes was stained with APC anti-mouse leukocyte

common antigen (CD45), PE/Cyanine7 anti-mouse integrin alpha X/ITGAX (CD11c), PE anti-mouse I-A/I-E, and anti-mouse cluster of differentiation 80 (CD80) antibodies. Infiltrating $CD8^+$ T cells in mesenteric lymph nodes were stained with anti-mouse CD45, FITC anti-mouse CD3, PerCP/Cyanine 5.5 anti-mouse CD8a, and PE anti-mouse FOXP3.

ELISAs of TNF-α, IFN-α, IFN-γ, and programmed death-ligand 1 (PD-L1) expression in tumors

Tumor tissues were homogenized and centrifuged, and the supernatant was collected for quantification of TNF-α, IFN-α, IFN-γ, and PD-L1 expression levels using ELISA kits.

Statistical analysis

The sample size calculation procedure, based on pilot experimental results and literature references, was detailed as follows.

For Sections (***F.n*** **drives TME remodeling via autophagy upregulation in CRC**, **Targeting intratumoral bacteria potentiates antitumor efficacy and remodels the immunosuppressive TME** ***in vivo***, **Eliminating intratumoral bacteria prevents tumor recurrence by stimulating long-term antitumor immunity**, and **Biosafety evaluation of HA@Ag NPs/PTX Cls**) sections (continuous variables), since tumor size and other metrics are continuous variables, we employed the following formula for continuous data:

$$n = \frac{2\left(Z_{\frac{\alpha}{2}} + Z_{\beta}\right) \times \left(Z_{\frac{\alpha}{2}} + Z_{\beta}\right) \times \delta^2}{\Delta^2}$$

Since the HA@Ag NPs/PTX Cls showed superior efficacy and the initial sample size calculation yielded an implausibly small value (n=0.37), we referred to prior literature to justify a final sample size of 5 per group [7,8].

For Section (**Targeting intratumoral bacteria potentiates antitumor efficacy and remodels the immunosuppressive TME** ***in vivo***) (categorical survival data), Fisher's exact test formula for categorical variables was used:

$$n = \frac{\left(Z_{\frac{\alpha}{2}}\sqrt{2\pi(1-\pi)} + Z_{\beta}\sqrt{p_1(1-p_1) + p_2(1-p_2)}\right)^2}{(p_1 - p_2)^2}$$

The computation yields a precise solution of n≈9. Ten mice per group were allocated for survival studies.

For Section (**HA-modified Cls exhibit superior tumor uptake *in vitro* and *in vivo***) (non-pharmacodynamic studies), including tumor targeting experiments and determination of the plasma concentration-time curve, we referred to prior literature to justify a final sample size of 3 per group based on established references [9,10].

For mechanistic validation (***F.n* drives TME remodeling via autophagy upregulation in CRC**), 20 mice were allocated (4 groups×5 mice/group). For the *in situ* tumor efficacy evaluation (**Targeting intratumoral bacteria potentiates antitumor efficacy and remodels the immunosuppressive TME *in vivo***), 35 mice were allocated (7 groups×5 mice/group). The survival study utilized 70 mice (7 groups×10 mice/group). In the rechallenge experiment (**Eliminating intratumoral bacteria prevents tumor recurrence by stimulating long-term antitumor immunity**), 15 mice were used (10 initially in the treatment group, with 5 long-term survivors, plus 5 controls). Safety evaluations (**Biosafety evaluation of HA@Ag NPs/PTX Cls**) included both acute and chronic toxicity studies, each employing 20 mice (2 groups×5 mice/group×2 studies). For non-pharmacodynamic studies, including targeting efficiency assessment and pharmacokinetic (PK) analysis, *in vivo* fluorescence retention imaging (**HA-modified Cls exhibit superior tumor uptake *in vitro* and *in vivo***), 9 mice were allocated (3 groups×3 mice/group). Biodistribution analysis employed 27 mice (3 groups×3 time points×3 mice/group). Pharmacokinetic evaluation utilized 30 mice (2 groups×5 time points×3 mice/group).

All experiments were conducted with the minimal required number of animals to achieve statistically significant results while complying with animal ethics guidelines.

References

1. Liu YH, Wu H, Wang SQ, Zhang XT, Gong LM, Xiao CC, *et al*. Biomimetic multifunctional nanodrugs enable regulating abnormal tumor metabolism and amplifying PDT-induced immunotherapy for synergistically enhanced tumor ablation. Mater Today. 2023;68:125-47.
2. Liu D, Liang S, Ma K, Meng QF, Li X, Wei J, *et al*. Tumor microenvironment-responsive nanoparticles amplifying STING signaling pathway for cancer immunotherapy. Adv Mater. 2024;36(6):e2304845.
3. Zhou H, Tang D, Kang X, Yuan H, Yu Y, Xiong X, *et al*. Degradable pseudo conjugated polymer nanoparticles with NIR-II photothermal effect and cationic quaternary phosphonium structural bacteriostasis for anti-infection therapy. Adv Sci. 2022;9(16):2200732.
4. Yu T, Guo F, Yu Y, Sun T, Ma D, Han J, *et al*. *Fusobacterium nucleatum* promotes chemoresistance to colorectal cancer by modulating autophagy. Cell. 2017;170(3):548-63.e16.
5. Xing X, Li XQ, Yin SQ, Ma HT, Xiao SY, Tulamaiti A, *et al*. OASL promotes immune evasion in pancreatic ductal adenocarcinoma by enhancing autolysosome-mediated degradation of MHC-I. Theranostics. 2025;15(6):2104-20.
6. Yamamoto K, Venida A, Yano J, Biancur DE, Kakiuchi M, Gupta S, *et al*. Autophagy promotes immune evasion of pancreatic cancer by degrading MHC-I. Nature. 2020;581(7806):100-5.
7. Wang M, Rousseau B, Qiu K, Huang G, Zhang Y, Su H, *et al*. Killing tumor-associated bacteria with a liposomal antibiotic generates neoantigens that induce anti-tumor immune responses. Nat Biotechnol. 2024;42(8):1263-74.
8. Chan JE, Pan CH, Rub J, Guzman G, Krause K, Brown E, *et al*. Critical role for a high-plasticity cell state in lung cancer. Nature. 2026;651(8104):231-41.
9. Zhao Y, Qin J, Yu D, Liu Y, Song D, Tian K, *et al*. Polymer-locking fusogenic liposomes for glioblastoma-targeted siRNA delivery and CRISPR-Cas gene editing. Nat Nanotechnol. 2024;19(12):1869-79.
10. Liu Y, Gong L, Feng J, Xiao C, Liu C, Chen B, *et al*. Co-delivery of axitinib and PD-L1 siRNA for the synergism of vascular normalization and immune checkpoint inhibition to boost anticancer immunity. J Nanobiotechnology. 2025;23(1):194.

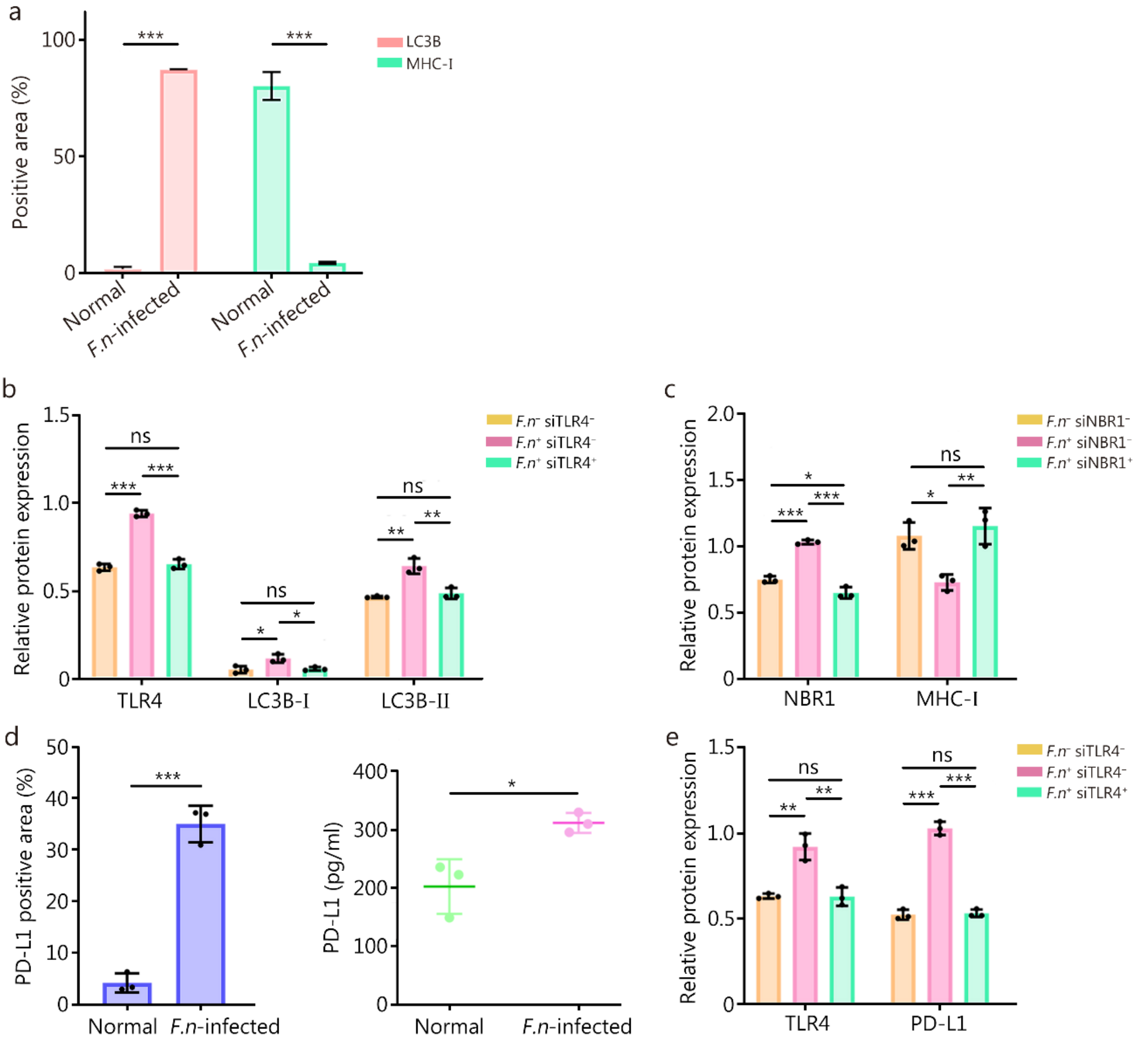

Fig. S1 Quantitative analysis of protein expression after *F.n*-infection. **a** LC3B and MHC-I expression were quantitatively analyzed by IHC staining of tumor tissues after *F.n*-infected. **b** Quantitative analysis of TLR4 and LC3B expression by western blotting. **c** Quantitative analysis of NBR1 and MHC-I expression by western blotting. **d** PD-L1 expression was quantitatively analyzed by IHC staining of tumor tissues and ELISA after *F.n*-infected. **e** Quantitative analysis of TLR4 and PD-L1 expression by Western blotting. Data were presented as mean±SD, analyzed by Student's *t*-test (**a**, **d**) and one-way analysis of variance followed by Tukey's post-test (**b-e**) (n=3). $^{*}P$<0.05, $^{**}P$<0.01, $^{***}P$<0.001, ns non-significant. MHC-I. Major histocompatibility complex class I; TLR4. Toll-like receptor 4; LC3B. Microtubule-associated protein 1A/1B-light chain 3B; NBR1. Neighbor of the BRCA1 gene 1; MHC-I. Major histocompatibility complex class I; PD-L1. Programmed death-ligand 1; IHC. Immunohistochemical; ELISA. Enzyme-linked immunosorbent assay; *F.n*. *Fusobacterium nucleatum*; SD. Standard deviation

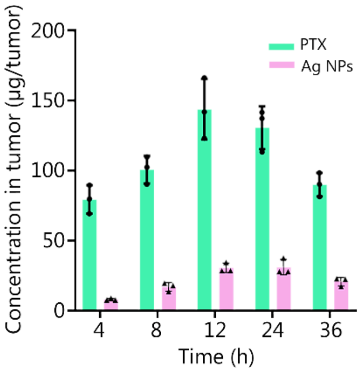

Fig. S2 The content of PTX and Ag NPs in tumor tissue after oral administration with HA@Ag NPs/PTX Cls at 4, 8, 12, 24, 36 h (*n*=3). Data were presented as mean±SD. HA. Hyaluronic acid; Cls. Cationic liposomes; PTX. Paclitaxel; Ag NPs. Silver nanoparticles; SD. Standard deviation

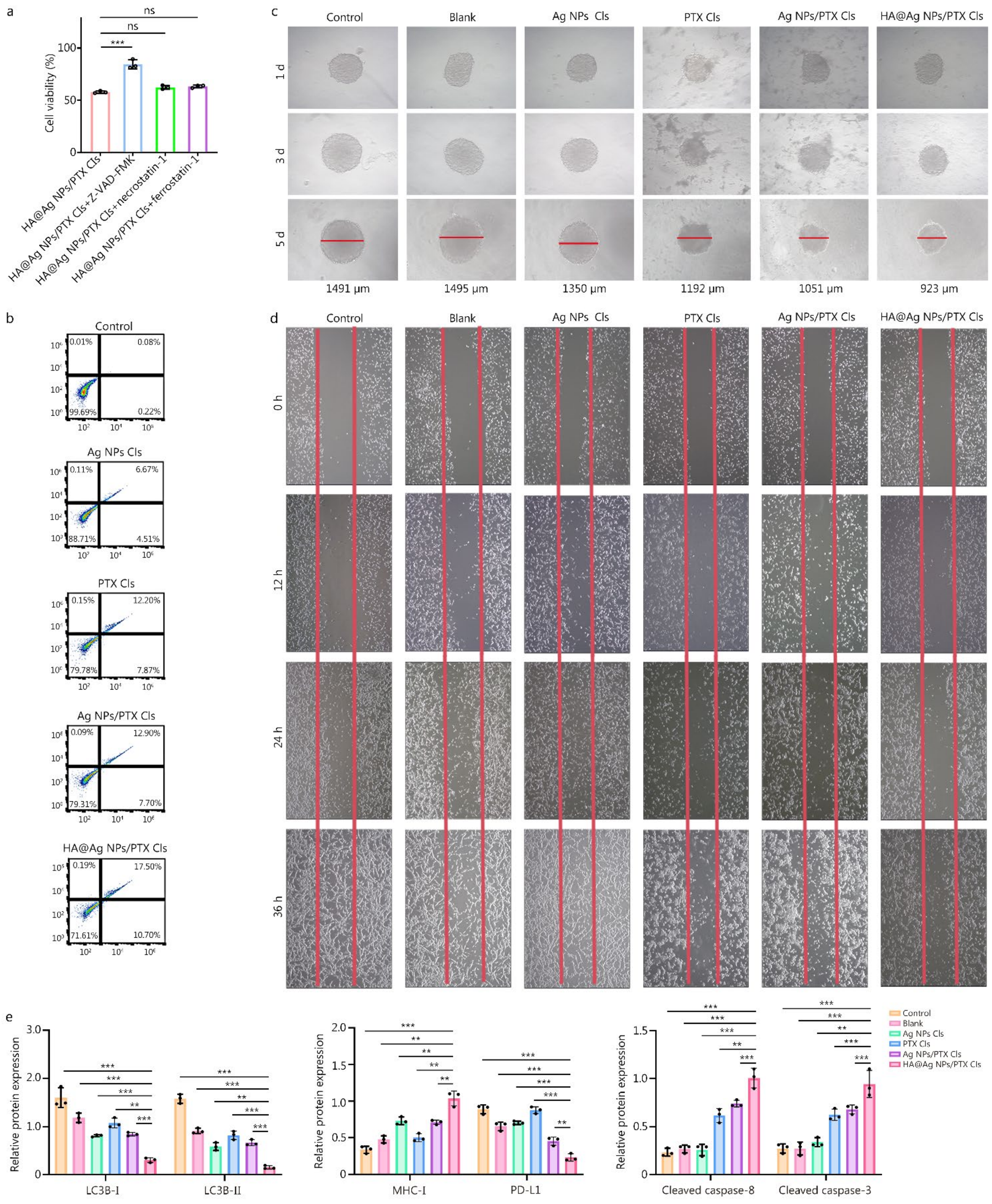

Fig. S3 Verification of cell death modalities and *in vitro* antitumor efficacy of the nanotherapeutics. **a** Verification of tumor cell apoptosis mode (*n*=3). **b** Cell apoptosis induced by various therapies tested by flow cytometry. **c** Comparison of the size of tumor spheres after various therapies. **d** The delineated area of tumor cells after being treated with various therapies. **e** Western blotting quantitative analysis of proteins involved in the autophagy pathway, MHC-I, PD-L1, and proteins involved in the apoptosis pathway in C26 cells. Data were presented as mean±SD,

analyzed by one-way ANOVA followed by Tukey's post-test (**a**, **e**). $^{**}P<0.01$, $^{***}P<0.001$, ns non-significant. HA. Hyaluronic acid; Ag NPs. Silver nanoparticles; PTX. Paclitaxel; Cls. Cationic liposomes; SD. Standard deviation; Z-VAD-FMK. Z-Val-Ala-Asp(OMe)-fluoromethylketon

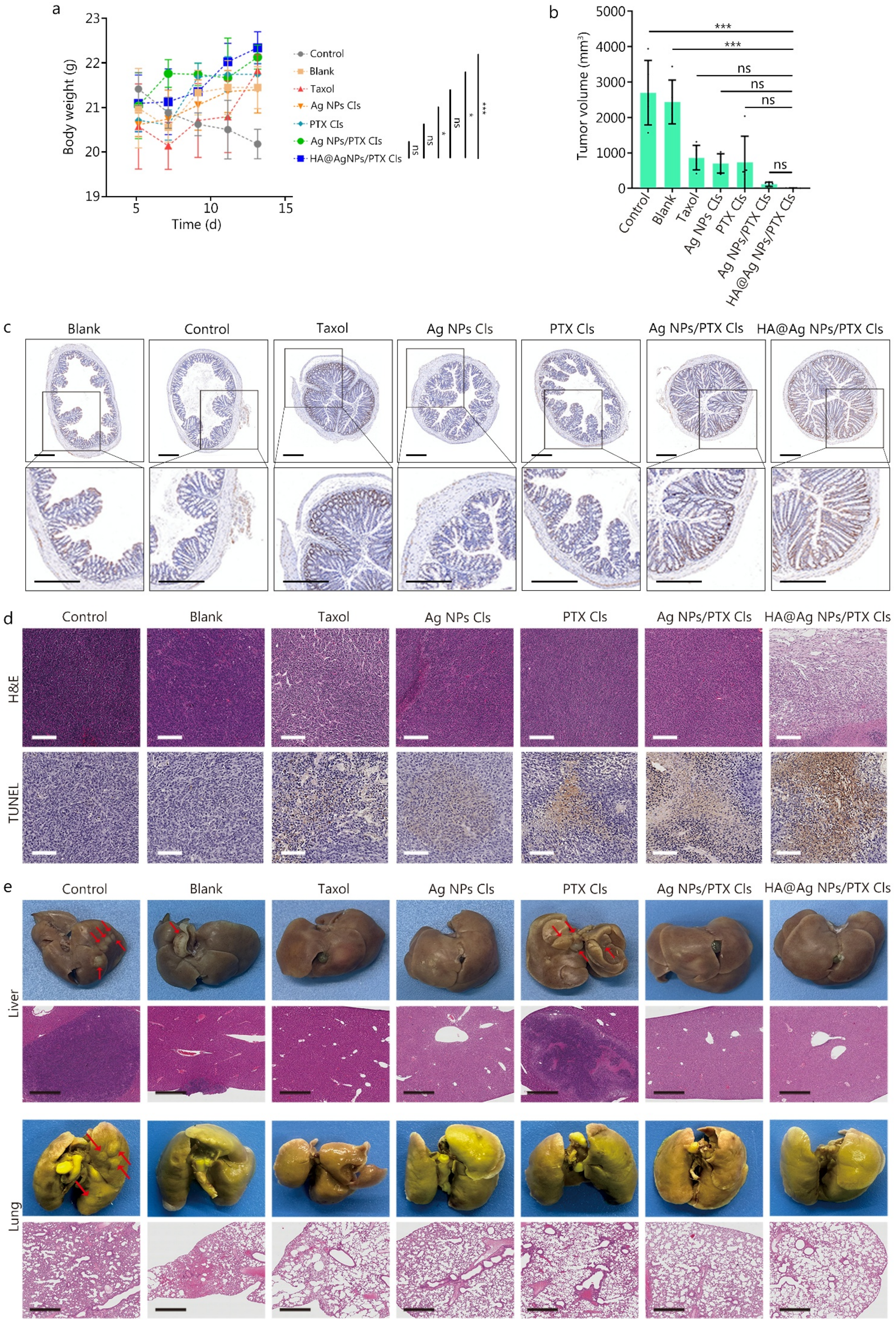

a
Body weight (g)
Time (d)
Control
Blank
Taxol
Ag NPs CIs
PTX CIs
Ag NPs/PTX CIs
HA@AgNPs/PTX CIs
ns
ns
*
ns
*
***
b
Tumor volume (mm³)
***
***
ns
ns
ns
ns
Control
Blank
Taxol
Ag NPs CIs
PTX CIs
Ag NPs/PTX CIs
HA@Ag NPs/PTX CIs
c
Blank
Control
Taxol
Ag NPs CIs
PTX CIs
Ag NPs/PTX CIs
HA@Ag NPs/PTX CIs
d
Control
Blank
Taxol
Ag NPs CIs
PTX CIs
Ag NPs/PTX CIs
HA@Ag NPs/PTX CIs
H&E
TUNEL
e
Control
Blank
Taxol
Ag NPs CIs
PTX CIs
Ag NPs/PTX CIs
HA@Ag NPs/PTX CIs
Liver
Lung

Fig. S4 *In vivo* antitumor efficacy of nanotherapeutics. **a** Body weight of mice during therapy (*n*=5). **b** Tumor volume at the end of therapy (*n*=5). **c** Intestinal Ki67 immunohistochemical staining at the end of therapy. Scale bar=500 μm. **d** H&E, TUNEL staining of tumor tissues after various therapies. Scale bar=200 μm. **e** Representative photographs and H&E staining of lung tissues and liver tissues after various therapies. Scale bar=500 μm. Data were presented as mean±SD, analyzed by one-way ANOVA followed by Tukey's post-test (**a**, **b**). $^{*}P<0.05$, $^{***}P<0.001$, ns non-significant. HA. Hyaluronic acid; Ag NPs. Silver nanoparticles; PTX. Paclitaxel; Cls. Cationic liposomes; Ki67. Kiel-67 antigen; H&E. Hematoxylin and eosin; TUNEL. Terminal deoxynucleotidyl transferase-mediated dUTP nick-end labeling; SD. Standard deviation

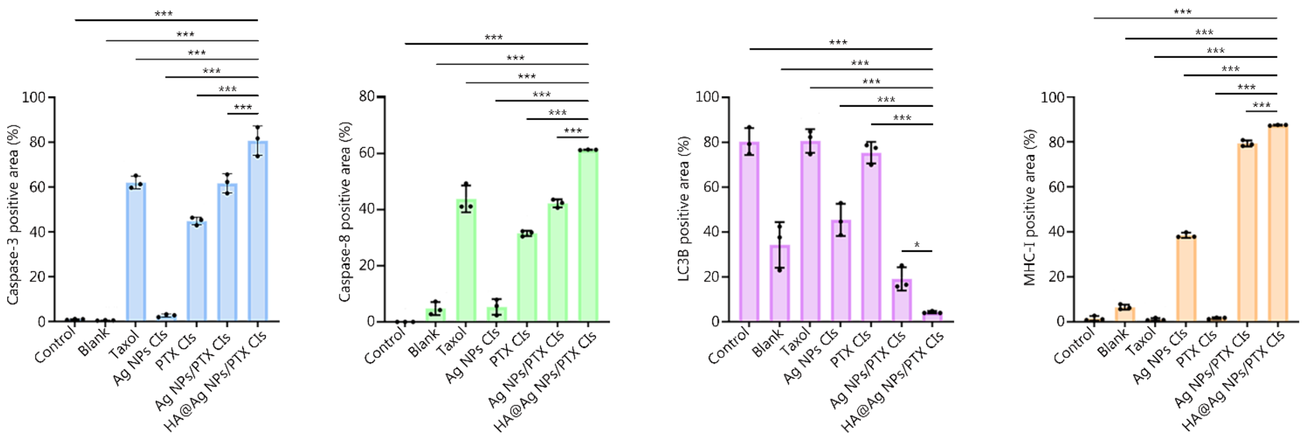

Fig. S5 Key markers expression in apoptosis pathway, autophagy pathway, and MHC-I expression were quantitatively analyzed by IHC staining of tumor tissues after treatment with different therapies (n=3). Data were presented as mean±SD, analyzed by one-way ANOVA followed by Tukey's post-test. $^{***}P<0.001$. HA. Hyaluronic acid; Ag NPs. Silver nanoparticles; PTX. Paclitaxel; Cls. Cationic liposomes. LC3B. Microtubule-associated protein 1A/1B-light chain 3B; MHC-I. Major histocompatibility complex class I; SD. Standard deviation

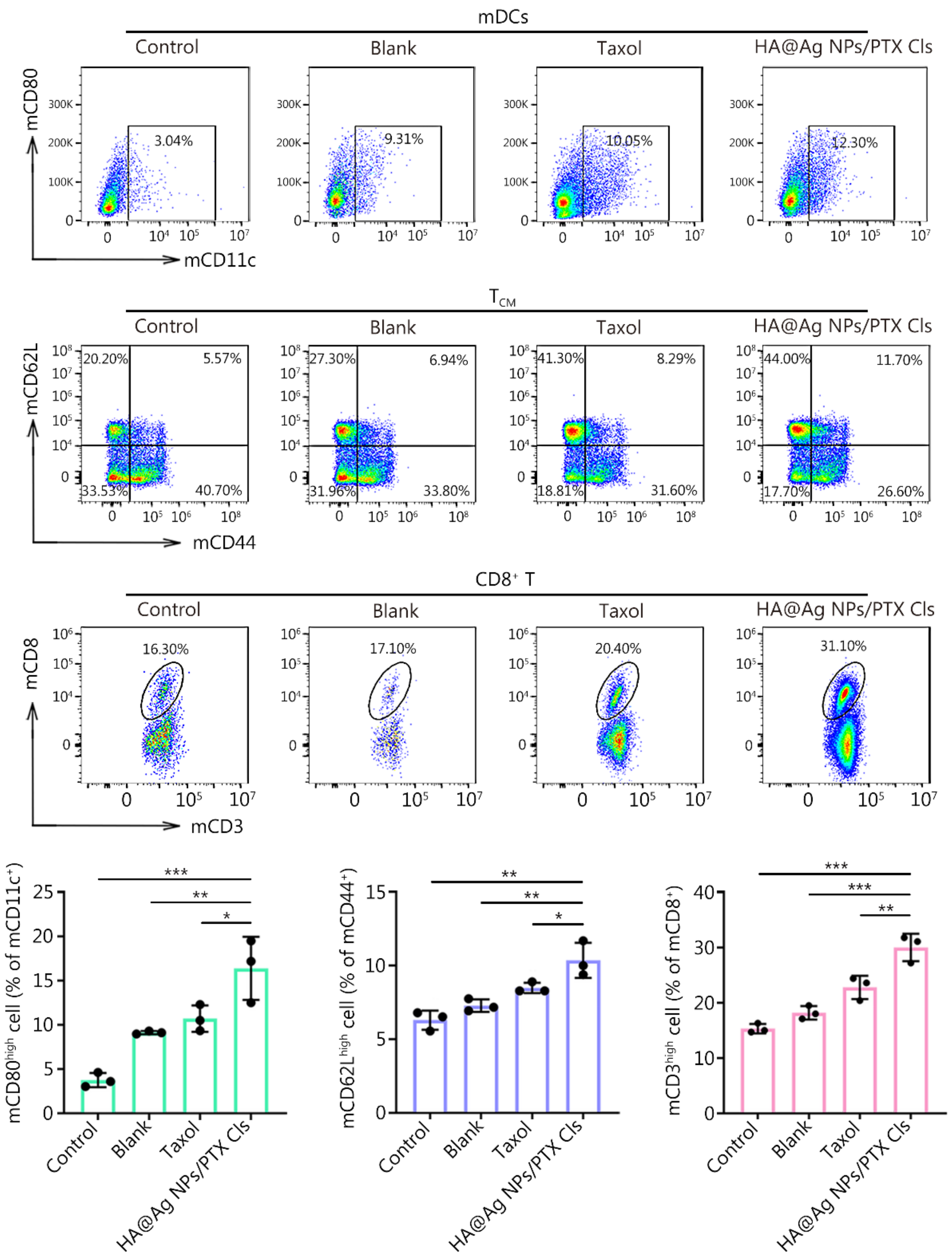

Fig. S6 Flow cytometry analysis of T_{CM} in spleen, mature DCs in mesenteric lymph node, and CD8$^+$ T cells in tumor tissues (*n*=3). Data were presented as mean±SD, analyzed by one-way ANOVA followed by Tukey's post-test. $^*P$<0.05, $^{**}P$<0.01, $^{***}P$<0.001. HA. Hyaluronic acid; Ag NPs. Silver nanoparticles; PTX. Paclitaxel; Cls. Cationic liposomes; DC. Dendritic cell; CD8. Cluster of differentiation 8; CD44. Cluster of differentiation 44; CD3. Cluster of differentiation 3; CD80. Cluster of differentiation 80; CD62L. Cluster of differentiation 62L; SD. Standard deviation; T_{CM}. Central memory T cells

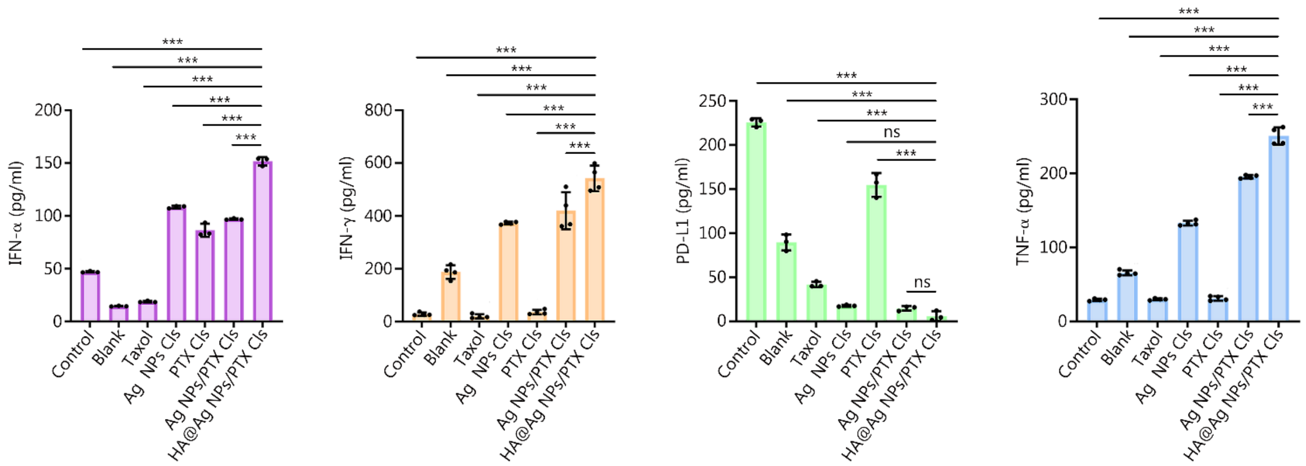

Fig. S7 Cytokine levels of IFN-α, IFN-γ, PD-L1, and TNF-α in tumor tissue were detected through ELISA among different groups (IFN-α and PD-L1: *n*=3, IFN-γ and TNF-α: *n*=4). Data were presented as mean±SD, analyzed by one-way ANOVA followed by Tukey's post-test. $^{***}P<0.001$, ns non-significant. HA. Hyaluronic acid; Ag NPs. Silver nanoparticles; PTX. Paclitaxel; Cls. Cationic liposomes; IFN. Interferon; PD-L1. Programmed death-ligand 1; TNF-α. Tumor necrosis factor-α; SD. Standard deviation; ELISA. Enzyme-linked immunosorbent assay

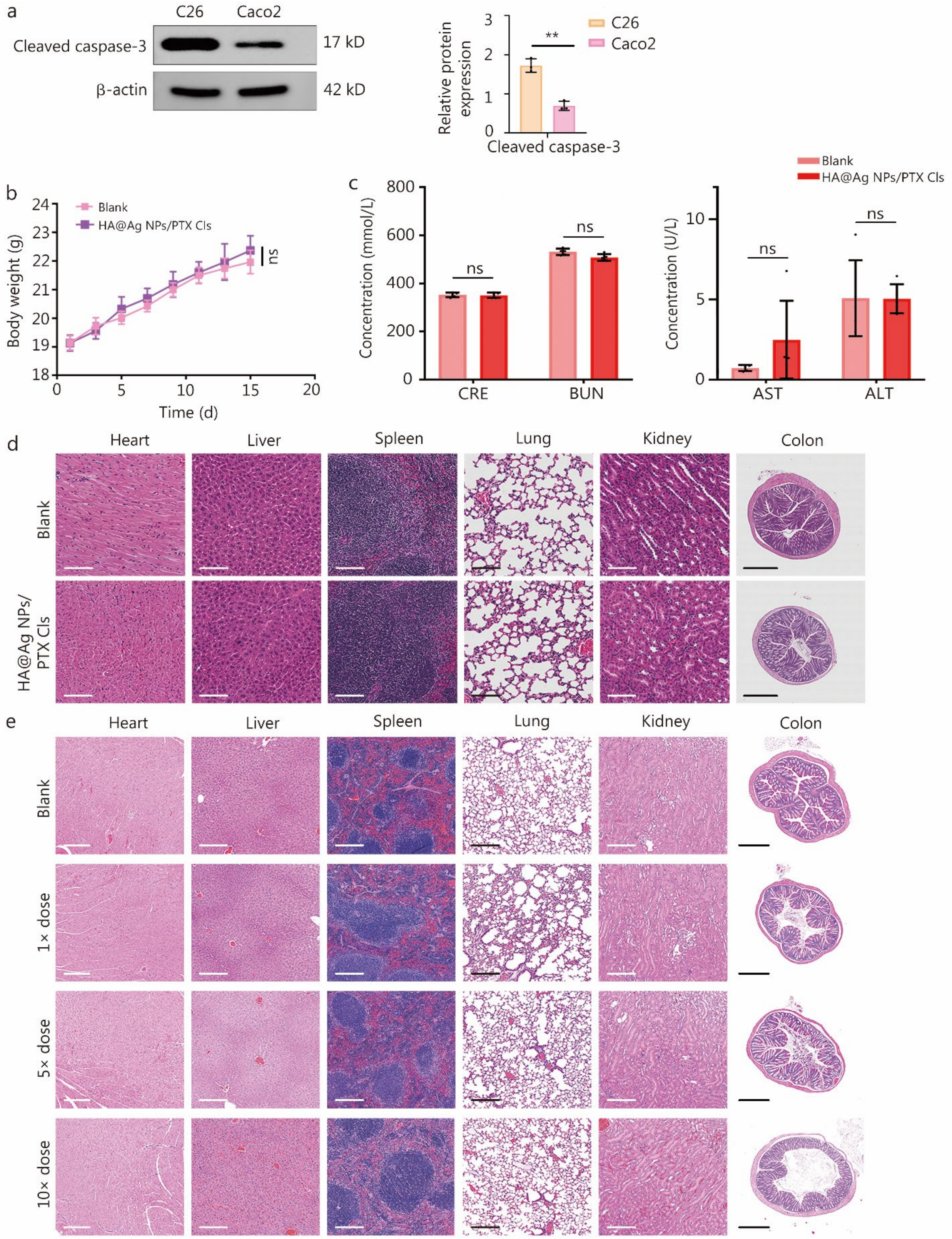

Fig. S8 Biosafety assessment of nanomedicine. **a** Western blotting and quantification on the apoptosis pathway in C26 cells and Caco2 cells incubated with HA@ Ag NPs/PTX Cls (*n*=3). **b** Body weight of mice during various therapies (*n*=5). **c** CRE, BUN, AST, and ALT levels of the blood from mice after treatment with different therapies (*n*=3). **d** The H&E staining images of main organs [heart, liver, spleen, lung, kidney (scale bar=200 μm), and colon (scale bar=500 μm)] of mice at the end of the acute toxicity test. **e** The H&E staining images of main organs [heart,

liver, spleen, lung, kidney (scale bar=200 μm), and colon (scale bar=500 μm) of mice at the end of the long-term toxicity test. Data were presented as mean±SD, analyzed by one-way analysis of variance followed by Tukey's post (**a**) and Student's *t*-test (**b**, **c**). ns non-significant. HA. Hyaluronic acid; Ag NPs. Silver nanoparticles; PTX. Paclitaxel; Cls. Cationic liposomes; CRE. Creatinine; BUN. Blood urea nitrogen; AST. Aspartate aminotransferase; ALT. Alanine aminotransferase; SD. Standard deviation; H&E. Hematoxylin and eosin

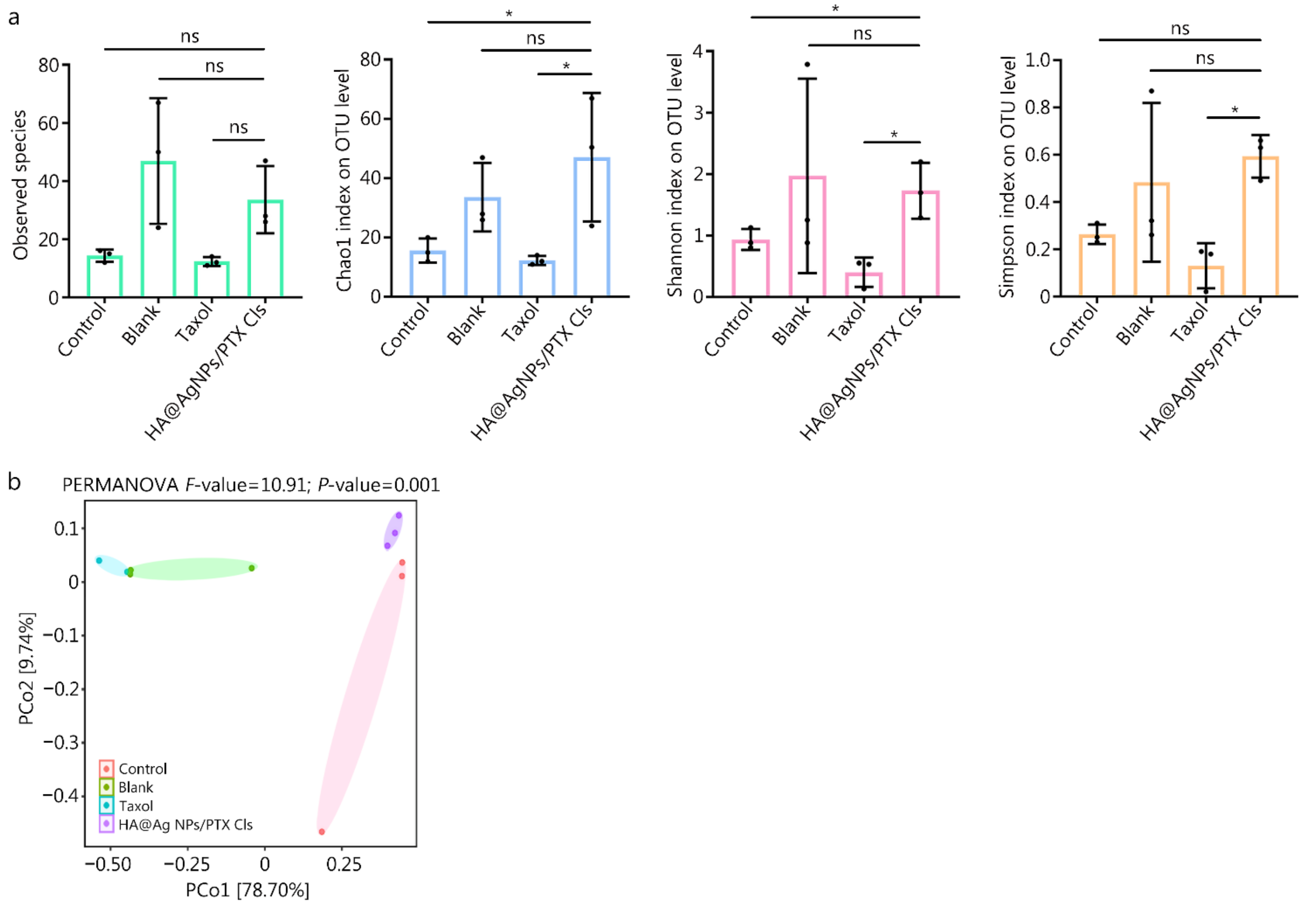

Fig. S9 Microbiome diversity analysis of intratumoral bacteria, alpha-diversity and beta-diversity. **a** Analysis of α-diversity coefficient intratumoral bacteria, OTU richness, Chao1 index, Shannon's index, and Simpson's index (n=3). **b** Perform principal coordinate analysis (PCoA) for β-diversity (n=3). Data were presented as mean±SD, analyzed by one-way ANOVA followed by Tukey's post-test (**a**) and PERMANOVA (**b**). $^{*}P$<0.05, ns non-significant. HA. Hyaluronic acid; Ag NPs. Silver nanoparticles; PTX. Paclitaxel; Cls. Cationic liposomes; OUT. Operational taxonomic unit; SD. Standard deviation; PERMANOVA. Permutational MANOVA

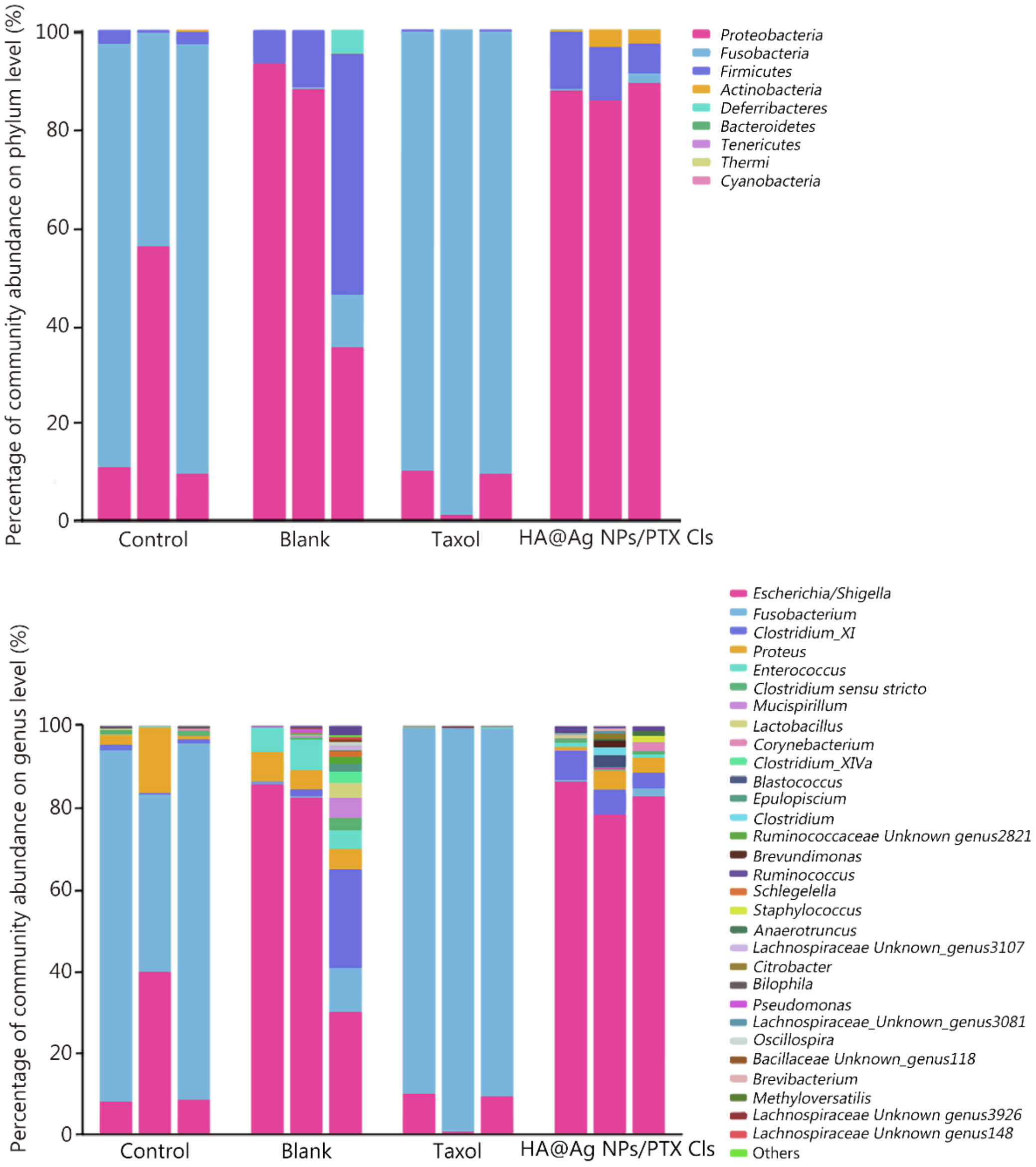

Fig. S10 Stacked histograms of the relative abundance of bacterial communities at the phylum and genus level in the indicated samples. HA. Hyaluronic acid; Ag NPs. Silver nanoparticles; PTX. Paclitaxel; Cls. Cationic liposomes

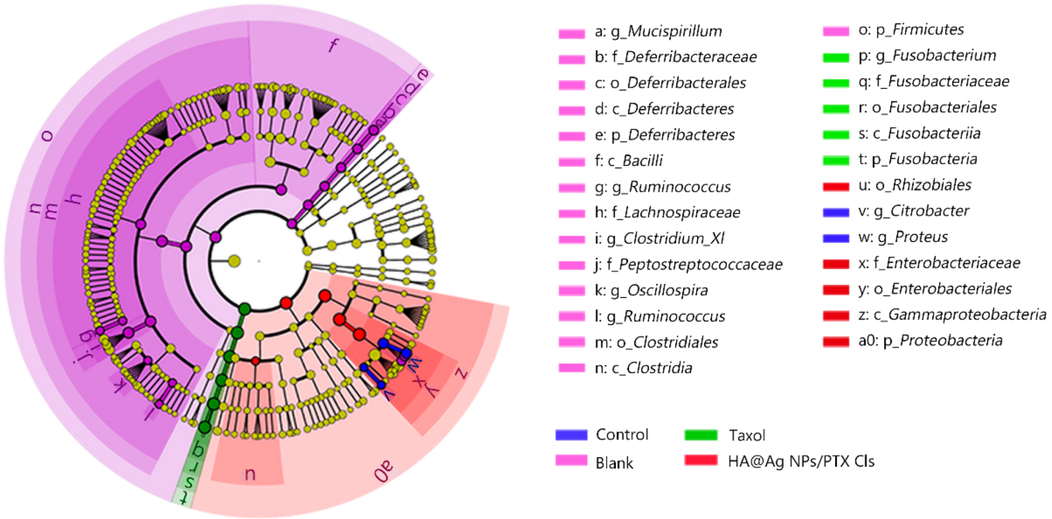

Fig. S11 Taxonomic branching map of bacteria in tumors after different groups of treatments, depicting taxonomic associations between microbial communities in tumors. Each node represents a specific taxonomic type. Yellow nodes indicate taxonomic features that are not significantly different between groups. Other colored nodes indicate taxonomic types with higher abundance in each group than others. HA. Hyaluronic acid; Ag NPs. Silver nanoparticles; PTX. Paclitaxel; Cls. Cationic liposomes

Table S1 Determination of Ag NPs encapsulation efficiency

Sample	Abs (410 nm)	Free Ag NPs content (μg/ml)	Free Ag NPs content (μg/ml, mean±SD)	Total Ag NPs (μg/ml)	EE% (mean±SD)
1	0.22	2.25			
2	0.26	2.69	2.19±0.64	10.00	(78.13±0.05)%-
3	0.16	1.63			

Standard curve: y=0.0947x+0.0047, *r*=0.9951. HA. Hyaluronic acid; Ag NPs. Silver nanoparticles; Abs. Absorbance; EE. Encapsulation efficiency; SD. Standard deviation

Table S2 Determination of PTX encapsulation efficiency

Sample	Area of Free PTX	Free PTX content (μg/ml)	Free PTX content (μg/ml, mean±SD)	Total PTX (μg/ml)	EE% (mean±SD)
1	0.10	1.03			
2	0.17	1.75	1.68±0.62	100.00	(99.98±0.01)%
3	0.22	2.26			

PTX. Paclitaxel; EE. Encapsulation efficiency; UV. Ultraviolet; HPLC. High-performance liquid chromatography

Table S3 Calculation of HA coating efficiency

Sample	Abs (450 nm)	Free HA content (ng/ml)	Free HA content (μg/ml, mean±SD)	Total HA (μg/ml)	EE% (mean±SD)
1	0.16	0.02			
2	0.17	0.03	0.02±0.01	10,000.00	(99.99±0.01)%
3	0.15	0.01			

Standard curve: $y=-0.0005x^2+0.0505x + 0.1395$, r=0.9999. Coating efficiency: 100.00%. HA. Hyaluronic acid; Abs. Absorbance; SD. Standard deviation

Table S4 Comparison of C6 fluorescence uptake among groups at 4 h

**Sample**	**Free C6**	**C6 Cls**	**HA@ C6 Cls**
1	3419	4737	7405
2	3394	4756	7198
3	3305	4832	7325
Average	3373	4775	7309

HA@ C6 Cls/C6 Cls=1.53; HA@ C6 Cls/Free C6=2.16. HA. Hyaluronic acid; Cls. Cationic liposomes; C6. Coumarin 6

Table S5 Comparison of DiR fluorescence in tumor tissue at 48 h

**Sample**	**DiR Cls**	**HA@ DiR Cls**
1	342,000,000	1,320,000,000
2	337,000,000	695,000,000
3	425,000,000	1,130,000,000
Average	368,000,000	1,048,333,333

HA@ DiR Cls/DiR Cls=2.85. HA. Hyaluronic acid; Cls. Cationic liposomes; DiR. 1,1'-dioctadecyl-3,3,3',3'-tetramethylindotricarbocyanine iodide
